# lncAPNet enables the deciphering of lncRNA–mRNA connections in patient transcriptomic data

**DOI:** 10.1093/bioadv/vbag204

**Published:** 2026-07-21

**Authors:** Vasileios Vasileiou, George I Gavriilidis, Pedro Faria Zeni, Marek Mraz, Evangelos Karatzas, Antonis Giakountis, Georgios A Pavlopoulos, Antonis Giannakakis, Fotis Psomopoulos

**Affiliations:** Institute of Applied Biosciences, Center for Research and Technology Hellas, Thessaloniki, 57001, Greece; Department of Molecular Biology and Genetics, Democritus University of Thrace, Alexandroupolis, 68100, Greece; Institute of Applied Biosciences, Center for Research and Technology Hellas, Thessaloniki, 57001, Greece; Center of Digital Health, Berlin Institute of Health at Charité - Universitätsmedizin Berlin, Berlin, 10117, Germany; Molecular Medicine, Central European Institute of Technology, Masaryk University, Brno, 625 00, Czech Republic; Molecular Medicine, Central European Institute of Technology, Masaryk University, Brno, 625 00, Czech Republic; European Molecular Biology Laboratory, European Bioinformatics Institute (EMBL-EBI), Cambridge, CB10 1SD, UK; Department of Biochemistry and Biotechnology, University of Thessaly, Larissa, 41500, Greece; Institute for Fundamental Biomedical Research, BSRC “Alexander Fleming”, Vari, 16672, Greece; Department of Computational Biology, Mohamed bin Zayed, University of Artificial Intelligence (MBZUAI), Abu Dhabi, PO Box 7909, United Arab Emirates; Department of Molecular Biology and Genetics, Democritus University of Thrace, Alexandroupolis, 68100, Greece; University Research Institute of Maternal and Child Health and Precision Medicine, National and Kapodistrian University of Athens, Athens, 11527, Greece; Institute of Applied Biosciences, Center for Research and Technology Hellas, Thessaloniki, 57001, Greece

## Abstract

**Motivation:**

Long non-coding RNAs regulate gene expression through chromatin remodeling, transcriptional control, and post-transcriptional modulation, influencing physiological cell homeostasis but also disease onset. Yet most transcriptomic and network-based studies rely on descriptive linear co-expression analyses, missing nonlinear and mechanistic insights. Emerging ML/DL methods offer promise but remain limited by data sparsity, noise, insufficient biological priors, and poor interpretability, constraining systems-level lncRNA-mRNA motif discovery.

**Results:**

In this manuscript, we introduce lncAPNet, an extended version of the APNet workflow, which integrates graph-based nonlinear inference of lncRNA–mRNA interactions using NetBID2’s and scMINERs activity logic within a lncRNA-focused SJARACNe co-expression network, coupled with PASNet, a biologically informed sparse deep learning model. This framework enables explainable identification of lncRNA drivers in three different cancer type case studies, two with bulk RNA-seq datasets [Chronic Lymphocytic Leukemia and Prostate Adenocarcinoma] and one by combining bulk RNA-seq and scRNA-seq omics datasets [Breast Invasive Carcinoma], uncovering lncRNA drivers that illuminate lncRNA-mediated programs in cancer progression.

**Availability and implementation:**

lncAPNet’s R scripts, Python scripts, and Nextflow pipeline are available at the GitHub repository: https://github.com/BiodataAnalysisGroup/lncAPNet.

## 1 Introduction

Long non-coding RNAs (lncRNAs) are a diverse class of RNA molecules, arbitrarily defined as longer than 200 nucleotides, that play crucial roles in regulating gene expression and various cellular processes (Mattick *et al.* 2023). They function as signal drivers, decoys, guides, scaffolds, or enhancers to control transcriptional and post-transcriptional events (Zeni and Mraz 2021). Functionally, lncRNAs guide chromatin-modifying complexes to specific genomic loci for histone or DNA methylation, thereby influencing gene silencing or activation. They also regulate transcription by modulating RNA polymerase activity, post-transcriptional processes like splicing and mRNA stabilization, and translation by interacting with ribosomes or initiation factors. Moreover, some lncRNAs influence post-translational modifications by affecting protein acetylation or phosphorylation. Through these mechanisms, lncRNAs maintain genomic integrity and regulate cell differentiation, development, and signalling pathways. Dysregulation of lncRNA expression has been increasingly linked to the initiation and progression of various cancers, highlighting their potential as biomarkers and therapeutic targets in oncology (Nandwani *et al*. 2021).

Transcriptomic studies of long non-coding RNAs (lncRNAs) primarily rely on bioinformatics approaches, with Differential Expression Analysis (DEA) and lncRNA-mRNA databases as the central strategy (Gong *et al.* 2021). However, they often remain descriptive, providing associations without directly uncovering the underlying molecular or regulatory functions of lncRNAs-mRNAs interactions or lncRNA-pathway-related mechanisms.

Current lncRNA-mRNA network-level interactions approaches primarily rely on co-expression correlations when constructing lncRNA-mRNA networks using the Weighted Correlation Network Analysis (WGCNA) algorithm. Notably, many *in silico* studies suggest the importance of the correlation of lncRNAs with mRNA-pathway correlations (Xie Y *et al*. 2023). In contrast, some computational studies (Hou *et al.* 2022) validated their computational results *in vitro*. However, due to the size of large-scale datasets and limited computational power, WGCNA cannot analyse all gene pairs and cannot capture nonlinear relationships between lncRNAs and mRNAs.

Despite their importance, the identification of lncRNAs in systems-level biological models remains limited. The majority of ML/DL models in the field, despite the ability to learn complex, non-linear, high-dimensional data patterns (Kourou *et al.* 2021), are not designed to capture the regulatory complexities or network-level behaviors of lncRNAs, nor do they incorporate biological priors essential for understanding their functional context (Kim and Lee 2023).

Many Deep Learning approaches are being tested to discover these nascent motifs underlying lncRNAs and other omics modalities (Gong *et al.* 2021). Despite its potential, deep learning in lncRNA research faces major limitations, including data scarcity, high noise, and heterogeneity that compromise model robustness and reproducibility. Furthermore, incomplete and inconsistent information in existing lncRNA databases, together with the “black box” nature of deep learning models and the computational complexity of multiomics data integration, severely constrain interpretability, feature extraction, and biological validation (Kim and Lee 2023).

Considering the above, we present lncAPNet, a novel computational pipeline that bridges a critical methodological gap in clinical bioinformatics: the lack of explainable, network-based frameworks tailored to long non-coding RNAs (lncRNAs). While existing deep learning approaches, including the recently reported APNet framework (Gavriilidis *et al.* 2025), successfully utilize transcriptomic data for patient classification, they are fundamentally optimized for protein-coding genes and fail to capture the complex, non-linear regulatory mechanics of non-coding elements. To resolve this architectural limitation, lncAPNet introduces three key innovations that expand the baseline APNet toolbox: first, it introduces lncRNA-associated regulons derived from information-theoretic activity analysis (NetBID2/scMINER) (Dong *et al.* 2023, Pan *et al.* 2025) to extract hidden non-coding signals; second, it embeds a specialized lncRNA knowledge graph (lncRNAlyzr-KG) directly into the biological priors of a sparse deep learning network (Evangelista *et al.* 2025); and third, it deploys a graph-based non-linear interpretation module to provide *post hoc*, explainable AI (XAI) insights into lncRNA-mRNA-pathways dynamics. We validated lncAPNet across three distinct cancer pilot cases revealing correlations with key clinical covariates: Chronic Lymphocytic Leukemia (CLL) and Prostate Adenocarcinoma (PRAD) utilizing bulk RNA-seq datasets, and Breast Invasive Carcinoma (BRCA) integrating both bulk and single-cell RNA-seq (scRNA-seq) datasets. In all three cohorts, the pipeline successfully captured ground-truth and novel lncRNA-mRNA pathway interactions, accurately mapping them to critical cancer hallmarks such as proliferation, metabolism, and tumour progression.

## 2 Methodology

### 2.1 Concise overview of the modular architecture of lncAPNet

**Figure 1:**
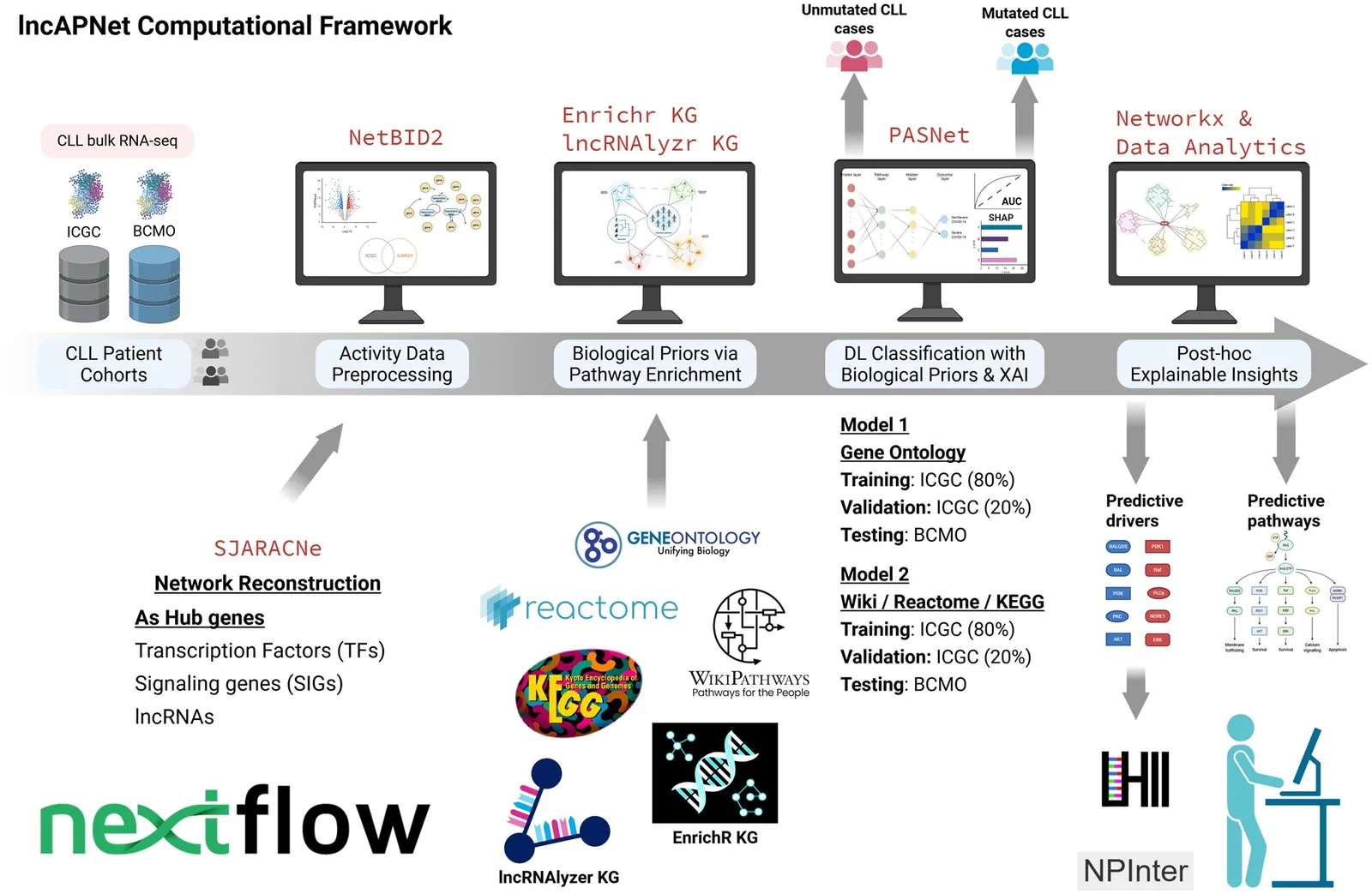
lncAPNet Overview. The lncAPNet workflow initially integrates NetBID2’s and scMINERs logic via the SJARACNe algorithm. Specifically, it identifies hub genes, including Transcription Factors (TFs), Signalling Genes (SIGs), and lncRNAs, and estimates their activity values using RNA-seq omics data. These activity values are then used as inputs to the PASNet algorithm, which leverages pathway information from GO, KEGG, Reactome, and WikiPathways as biological priors. Pathway–gene associations for mRNAs and lncRNAs are derived from the EnrichR-KG and lncRNAlyrs-KG databases developed by the Ma’ayan Lab, respectively. Finally, the critical features and pathways identified by PASNet are subjected to *post hoc* analysis using the open-source lncHUB2 tool to retrieve functional annotations for the relevant lncRNAs, while Kaplan–Meier plots are generated separately to identify potential biologically and prognostically meaningful drivers. Created with BioRender.com.

lncAPNet employs a three-stage pipeline. First, RNA-seq expression matrices are transformed into regulatory activity matrices using the SJARACNe/NetBID2-scMINER algorithm (Dong *et al.* 2023, Pan *et al.* 2025). Second, a biologically structured deep learning (DL) classifier based on the PASNet algorithm (Hao *et al.* 2018) is applied. Model explainability is achieved through SHAP (Shapley Additive Explanations) values (Aas *et al*. 2021), which highlight the most informative lncRNAs, mRNAs, and their associated pathways. In the final stage, lncRNAs of interest are identified, and the lncHUB2 Appyters tool (Evangelista *et al.* 2025) is used to extract detailed information about these features, while Kaplan-Meier plots are generated to estimate the survival probability over time for each potential driver (Fig. 1).

The key advancement of lncAPNet over the original APNet framework lies in its expanded initialization strategy: lncRNAs are designated as hub genes within the SJARACNe/NetBID2-scMINER algorithm, extending beyond the traditional focus on transcription factors (TFs) and signalling genes (SIGs). Additionally, lncAPNet incorporates pathway information linked to lncRNAs by leveraging the lncRNA-related knowledge graph developed by Ma’ayan’s Lab (lncRNAlyrs), thereby enriching the model’s biological context and interpretability. Finally, identified lncRNAs are validated using open-source annotated tools such as lncHUB2.

### 2.2 Activity values estimation and driver inferences

For data formatting, we ensured a consistent dataset by preprocessing it. In this automated step, the inputs (count matrix, metadata, and optionally feature information) were converted into an ExpressionSet object, containing a count matrix, sample metadata, and optionally feature annotations, enabling use of the NetBID2 and scMINER pipelines for analyses.

The ExpressionSet objects from the case studies used in this analysis were processed separately using the same workflow within the NetBID2 and scMINER frameworks. Count matrix converted to log_2_-formatted values to reduce data skewness and variance and prepare the data for subsequent statistical tests. Additional data preprocessing was also performed to discover medium-variability transcripts and informative samples for more detailed analysis.

**NetBID2 and scMINER** utilize the **SJARACNe** algorithm to reverse-engineer context-specific interactomes, and derive “activity values” for each candidate driver gene. We define Transcription Factors (TFs), Signalling Genes (SIGs) from MSigDB, and long non-coding RNAs (lncRNAs) retrieved from BioMart as the pool of potential drivers for SJARACNe.

SJARACNe (Khatamian *et al.* 2019) extends the original ARACNe method (Margolin *et al.* 2006) to handle large-scale data and assesses whether mutual information or correlation potentials are significantly different from zero. NetBID2 and scMINER then leverage_,_ the SJARACNe output to calculate driver “activity values” as the weighted mean of each driver’s target genes:


$${\mathrm{Driver}}_{si}=\;\frac{\sum_{j=1}^{n}({\mathrm{SIGN}}_{ij}\;\times \;{MI}_{ij}\;\times \;\;{\text{EXP}}_{sj})}{n}$$

where *EXPsj* is the expression value of the target protein *j* in sample *s*, *MIij* is the mutual information between driver *i* and target *j*, and *SIGNij* is the sign of their Spearman correlation. Finally, *n* is the number of targets regulated by *i*.

To account for the characteristic sparsity of the scRNA-seq dataset during scMINER analysis, target feature activity was calculated using the “getActivity_inBatch” function via the mean activity estimation method.

To identify statistically significant features in the TCGA bulk RNA-seq dataset, differential activity analysis was performed using the 'getDE.BID.2G' function from the NetBID2 package.

### 2.3 Biological priors

For each case scenario, statistically significant up- and down-regulated (adj-*P*value < .05) drivers were mapped to biological pathways using the gseapy Python package, with gene-pathway associations derived from **EnrichR KG** for mRNAs (Evangelista *et al.* 2023) and **lncrnalyzr KG** for lncRNAs (Evangelista *et al.* 2025). The considered databases for the pathways enrichment use the Gene Ontology (GO), KEGG, Reactome, and Wikipathways. Pathways that pass the statistical significance cutoff were then depicted as one-hot encodings, where genes were assigned 0 if absent from a given pathway and 1 if present. Notably, because of the large number of GO pathways identified from the enrichment analysis, the modeling was carried out in two separate training runs: the first incorporating only the GO pathways, and the second including all remaining databases.

### 2.4 Deep learning neural-network classification through PASNet-based biological priors

lncAPNet employs a sparse neural network, **PASNet** (Hao *et al.* 2018), for deep learning-based classification. PASNet integrates hierarchical driver-pathway relationships, allowing predictions to be both interpretable and explainable.

The mathematical foundation of PASNet involves enforcing layer-wise sparsity and incorporating cost-sensitive learning, which integrates hierarchical driver-pathway connections to produce more interpretable and explainable predictions. Specifically for the algorithmic background of PASNet:


$$h^{\left(l+1\right)}=\alpha ((W^{l}\;{}^{\circ}\;M^{\left(l\right)})h^{l}+\;b^{l})$$

where ° is elementwise multiplication. For imbalanced data, the cost function includes class-specific average errors *ck*. We optimize weights *W(l)* and biases *b(l)* via gradient updates:


$$W^{\left(l\right)}\;\leftarrow (1-\;\eta \lambda )W^{l}-\;\eta \;\sum_{k=1}^{K}\frac{\vartheta C_{k}}{\vartheta W^{\left(l\right)}},\;b^{\left(l\right)}\;\leftarrow \;b^{\left(l\right)}-\sum_{k=1}^{K}\frac{\vartheta C^{k}}{\vartheta b^{\left(l\right)}}$$

### 2.5 SHAP values

The **SHAP values** algorithm utilizes game theory to select the most interpretable feature drivers from the trained model. It computes a contribution score for each gene by quantifying its impact on the model prediction in every combination of drivers feasible, such that it gives a reasonable and consistent measure of importance. By adding up such contributions, drivers are ranked by their effect to gain insight into underlying biological processes and increase model interpretability. The mathematical rationale behind SHAP values is:


$$\varphi_{i}\;(f)=\sum_{S\subseteq N\{i\}}^{}\frac{\left|S|!(M-|S|-1\right)}{M!}\;[f(S\cup \{i\}\;-\;f(S))]$$

where f is the input model and the N the number of input features.

### 2.6 *Post hoc* explainability

Finally, lncAPNet offers diverse graph-based visualizations and analytical tools to extract meaningful biological insights. Feature importance is assessed using SHAP values, providing interpretable rankings of influential factors. Based on these rankings, the top predictive drivers can be selected to enhance the SJARACNe networks using PASNet driver-pathway correlations, thereby generating regulatory subgraphs that reveal key regulatory relationships. Specifically, lncAPNet can create driver-pathway bipartite graphs that combine multiple metrics (such as mutual information from SJARACNe-derived gene-gene correlations and PASNet learning gene-pathway weights), enabling researchers to filter and pinpoint subgraphs with potential translational significance. By blending these databases’ PASNet structural weights with the empirical, data-driven networks from SJARACNe, lncAPNet translates multi-layered network embeddings into concrete driver-pathway subgraphs. This synthesis enables the pipeline to validate purely statistical lncRNA-mRNA correlations against pre-existing, cross-referenced biochemical pathways. Ultimately, this unified framework bridges the gap between high-dimensional deep learning outputs and traditional mechanistic biology, providing a robust tool for target discovery.

To obtain additional information on the lncRNAs of interest, we used the lncHUB2 pipeline. Specifically, we utilized the lncHUB2 UMAP part to visualize lncRNA expression across 18 705 lncRNAs from ARCHS4 (Marino *et al.* 2023). As part of this process, the samples were first log2-transformed and quantile-normalized along the gene axis, after which UMAP was applied to the lncRNA expression data, treating samples as features. Moreover, the lncHUB2 resources provide a method to assess whether the lncRNAs of interest are expressed across various tissues and cell types using ARCHS4 data (Lachmann *et al.* 2018). Users can input specific lncRNAs along with tissues or cell types of interest, to assess whether these lncRNAs exhibit high expression across established tissues and cell types in databases. Finally, significant lncRNAs can be further analysed with Kaplan–Meier plots (via the lifelines Python package) to assess their association with patient overall survival, providing insights into their potential prognostic value. Although overall survival analysis is critical, it is optional due to certain datasets lacking clinical characteristics.

APNet was developed following the **DOME guidelines** (Walsh *et al.* 2021) for reporting supervised machine learning analyses in biological studies (see Data and Code Availability).

## 3 Results

### 3.1 Case studies

The three pilot studies differed in cancer type, design, and goals. The CLL study examined a hematologic cancer with heterogeneous outcomes, stratifying patients by IGHV status (Unmutated vs. Mutated) to capture prognostic differences. The PRAD study focused on a solid tumor and compared early (I–II) versus late (III–IV) clinical stages to assess progression. A third scenario utilized a breast invasive carcinoma (BRCA) dataset to compare good-prognosis Luminal A samples against poor-prognosis Basal samples, integrating single-cell RNA sequencing (scRNA-seq) data to focus specifically on gene expression within cancer epithelial cells. Both used lncAPNet, but the CLL study applied a cross-dataset PASNet approach with separate training and testing cohorts and incorporated survival data to identify prognostic lncRNAs, while the BRCA scenario employed scRNA-seq to validate cell-specific expression levels while also performing survival analysis on lncRNAs of interest. In contrast, the PRAD study evaluated a single cohort without a survival analysis and identified only stage-associated lncRNAs. Overall, the CLL study emphasized cross-cohort molecular drivers, the BRCA scenario validated cell-type-specific drivers activity, and the PRAD pilot identified stage-related drivers within a single dataset.

### 3.2 Case study 1: Chronic lymphocytic leukemia—CLL

#### 3.2.1 CLL Case study overview

**Figure 2:**
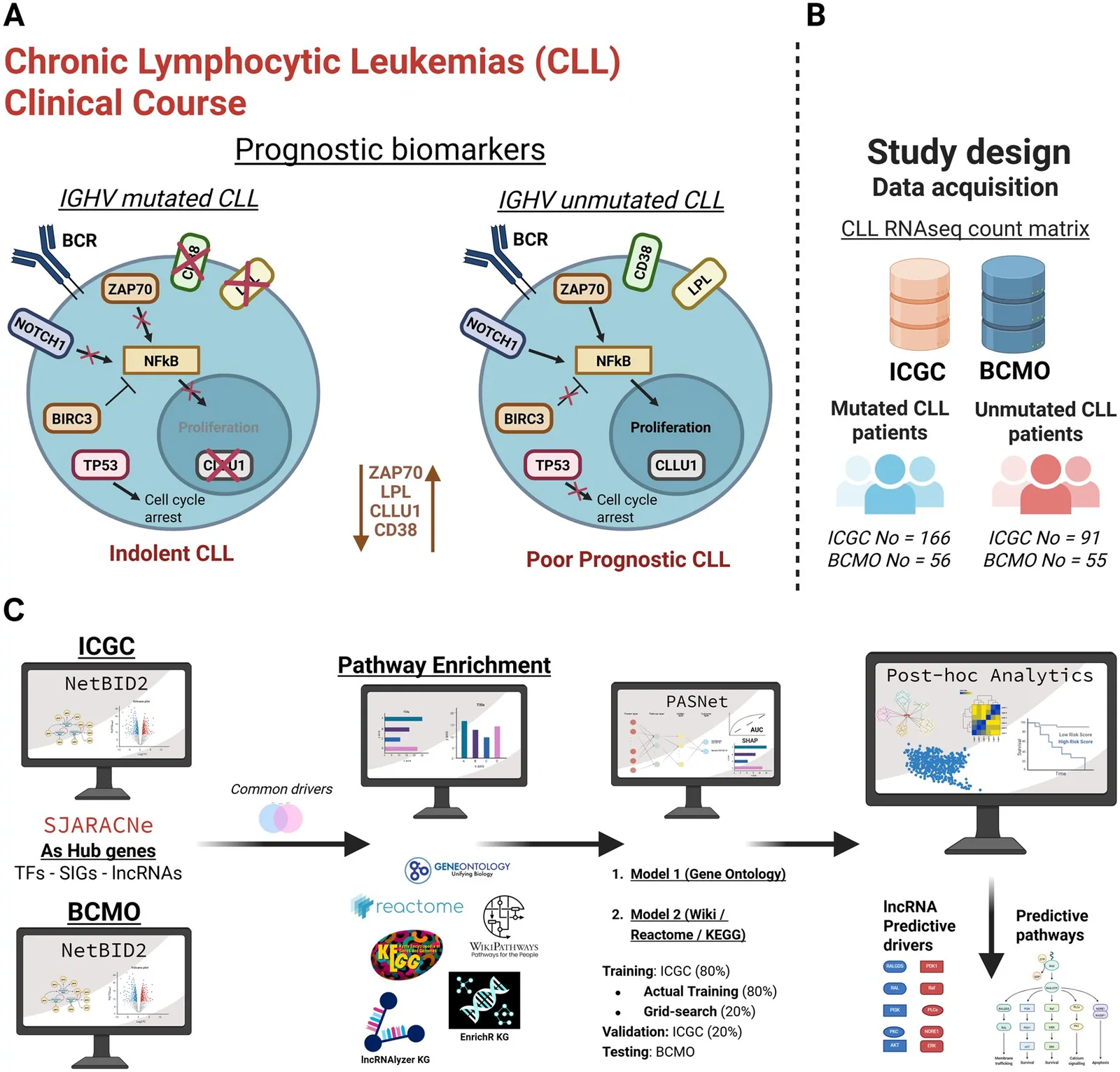
The lncAPNet framework applied to the CLL case study. (A) Brief explanation of the differences between Unmutated and Mutated CLL conditions, including their known clinical prognostic biomarkers, based on Rosenquist *et al*. (2013), and (B) a summary of the CLL patients in the dataset are stratified by IGHV status (U-CLL versus M-CLL). (C) Οverview of lncAPNet applied in the CLL case-study scenario. Created with BioRender.com.

The first pilot case of lncAPNet concerns CLL, one of the most common blood malignancies in the Western world. This leukemia is primarily dichotomized based on the mutational status of the IGHV gene in the B-cell receptor in indolent cases that require little to no treatment (Mutated-CLL, M-CLL) and in aggressive cases that can develop drug resistance with often fatal outcomes (Unmutated-CLL, U-CLL) (Fig. 2A) (Stevenson *et al.* 2011, Seda and Mraz 2015).

The primary objective of this analysis was to identify lncRNAs that differ in terms of activity between U-CLL and M-CLL cases. To ensure robustness, two independent datasets, the International Cancer Genome Consortium (ICGC under the project CLLE‑ES) and BloodCancerMultiOmics2017 Bioconductor database (BCMO) CLL cohorts, were initially analysed separately using NetBID2, with one dataset designated as the training set and the other as the testing set within the PASNet framework. Both datasets included survival data, enabling downstream survival analyses of the lncRNAs identified.

#### 3.2.2 Data retrieval and appropriate format creation

Bulk RNA-seq count matrices and their relevant clinical characteristics were first gathered from the ICGC and the BCMO (see Data Availability).

In the ICGC-CLL dataset, IGHV mutation status was manually extracted from the associated metadata provided by (Puente *et al.* 2015), whereas for the BCMO dataset, the mutation status was directly available in the relevant metadata (Dietrich *et al.* 2018).

The total number of samples for ICGC-CLL was 257 (split into 166 mutated and 91 unmutated IGHV status according to the study above), with a total amount of 38 687 annotated transcript IDs. The BCMO’s dataset had a total of 111 samples (split into 56 mutated and 55 unmutated IGHV status) with a total amount of 22 915 annotated transcript IDs (Fig. 2B).

##### 3.2.3 Activity analysis and identification of driver genes

We first applied the SJARACNe to network reconstruction using their relevant gene lists (TFs, SIGs, lncRNAs) and ran it separately for each dataset, using the default parameters of the SJARACNe algorithm (number of epochs at 100, consensus *P*-value threshold at 5 × 10^−8^).

Then, we used the NetBID2 algorithm to calculate gene activity and perform differential activity analysis independently on the ICGC and BCMO datasets. Differential analysis filtering was used to retain genes (adjusted *P*-value < .05 and target size > 30, suggested by the NetBID2 guidelines for robust driver inference). Specifically, for the ICGC dataset, 1921 drivers were identified, comprising 723 hyper-active and 1198 hypo-active genes (793 overt and 1070 hidden drivers), whereas in the BCMO dataset, 3551 significant drivers were identified, including 2164 hyper-active and 1387 hypo-active genes (1176 overt and 2261 hidden drivers). By categorizing drivers as positive (hyper-active) or negative (hypo-active) in each dataset, we identified 362 common positive and 212 common negative drivers, for a total of 574 shared drivers (Supplementary Fig. 1).

##### 3.2.4 Pathway enrichment analysis

To investigate the functional relevance of these drivers, pathway enrichment analysis was performed on the common hyperactive and hypoactive drivers using the GO, Reactome, KEGG, and Wikipathways databases. A significant cutoff of adjusted *P*-value < .05 was applied, resulting in the identification of 256, 103, 45, and 28 pathways, respectively.

Given the high number of GO pathways, we conducted two separate analyses/model training: (i) GO enrichment with 256 pathways, involving 229 of 574 drivers, and (ii) enrichment across KEGG, Reactome, and Wikipathways with 176 pathways, involving 269 of 574 drivers (Supplementary Fig. 2).

##### 3.2.5 Model training and validation

The ICGC dataset was partitioned into three subsets. First, an 80–20 split was applied to create the training and validation sets. The training portion (80%) was then further divided into training and grid-search validation sets using an 80–20 ratio. For hyperparameter tuning, the second training subset was used, and model performance was evaluated to determine the optimal learning rate (LR) and L2 regularization parameters over 10 000 epochs, and drop-out rates [0.8, 0.7]. These optimal parameters were then used for the final training, which was performed on the training subset and the dataset’s second validation subset. After model training, performance was evaluated on an independent test dataset, BCMO. These steps were followed for both models.

**Figure 3:**
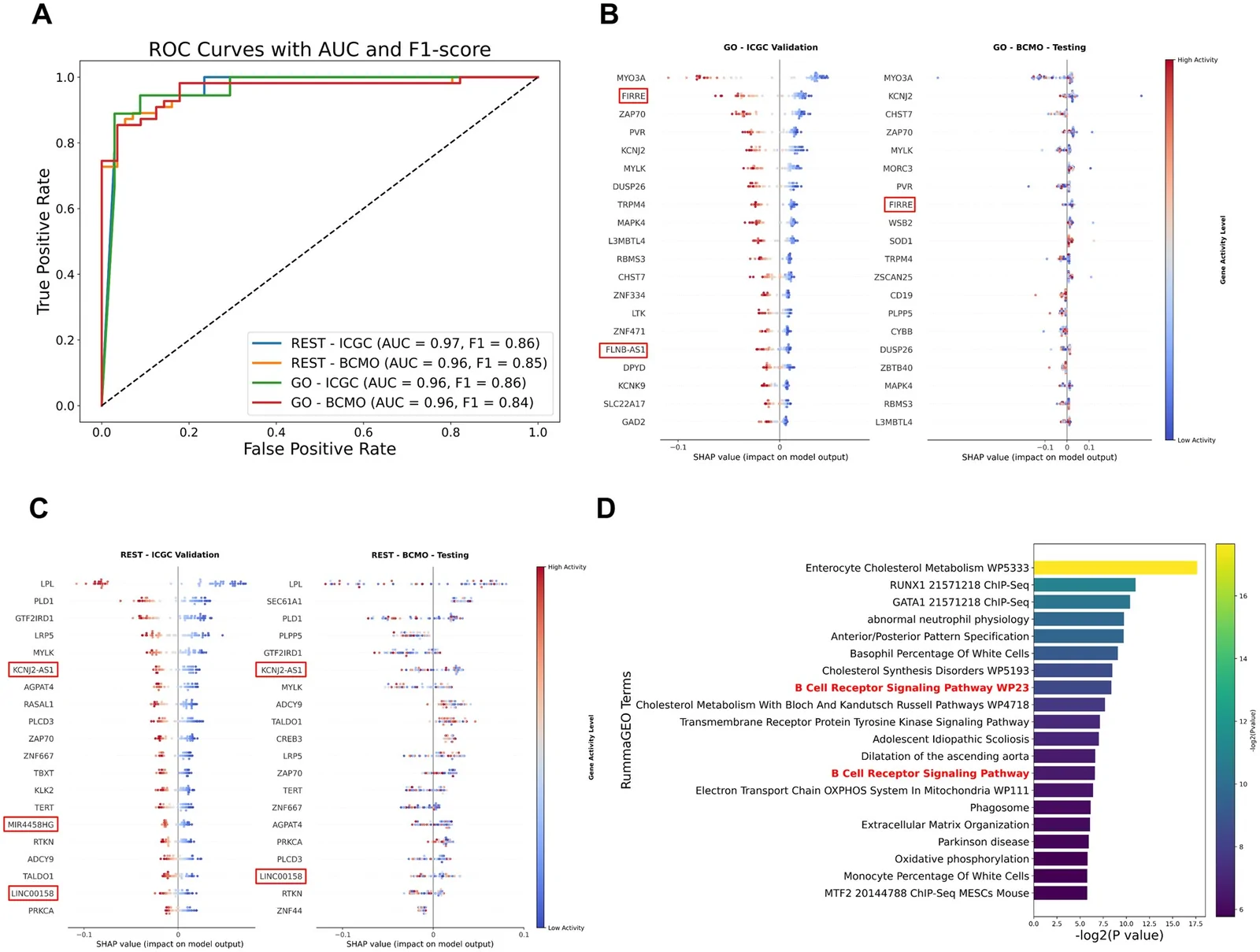
lncAPNet results in the CLL case study. (A) ROC curves with AUC and F1 scores for different lncAPNet configurations, including ICGC validation using the GO database, ICGC validation using additional databases, BCMO testing with the GO database, and BCMO testing with the remaining databases. (B, C) Top 20 SHAP features displayed as SHAP plots for each comparison scenario: ICGC validation database and BCMO testing with the GO database (B), ICGC validation with other databases, and BCMO testing with the rest databases (C). (D) Pathway enrichment analysis (RummaGEO) based on top SHAP features, with terms associated with both CLL and IGHV status highlighted in red on the *y*-axis. Created with BioRender.com.

For GO-based analysis, the ICGC-training and ICGC grid-search validation datasets yielded an optimal learning rate (LR = 0.005), L2 regularization (L2 = 0.0005), and drop-out rates [0.8, 0.7]. Subsequently, lncAPNet was trained and validated on the ICGC datasets, achieving robust performance metrics (AUC = 0.96, F1 score = 0.86). Applying this model to the independent BCMO dataset produced similarly high metrics (AUC = 0.96, F1 score = 0.84) (Fig. 3A).

For the combined KEGG, Reactome, and WikiPathways analysis, the optimal hyperparameters were LR = 0.007 and L2 = 0.0005. Training and validation on the ICGC datasets achieved an AUC of 0.97 and an F1 score of 0.86, while external validation on the BCMO dataset yielded an AUC of 0.96 and an F1 score of 0.84 (Fig. 3A).

##### 3.2.6 Identification of key lncRNA drivers

For each model, a SHAP analysis was conducted to identify the top 20 features contributing to its predictions (Fig. 3B and C). By combining the SHAP values from both models, 49 unique significant explainable drivers were identified, including five lncRNAs: FIRRE, MIR4458HG, LINC00158, KCNJ2-AS1, and FLNB-AS1. Enrichment analysis using RummaGEO demonstrated that those top 49 drivers were involved in BCR signaling (Fig. 3D).

#### 3.2.7 Bipartite graphs driver-pathway correlations

After extracting the driver-pathway correlations from PASNet, we exclude weights that fall within the interquartile range (25th–75th percentile) to focus on the most relevant correlations. We then integrated the outputs from both GO and other pathway databases (KEGG, Reactome, and WikiPathways) and applied a filtering criterion, retaining pathways associated with at least three drivers and drivers that participate in at least one pathway. Through this approach, we identified four lncRNAs [FIRRE, LINC00158, KCNJ2-AS1, and FLNB-AS1] that exhibited significant correlations with these pathways (Supplementary Fig. 3).

**Figure 4:**
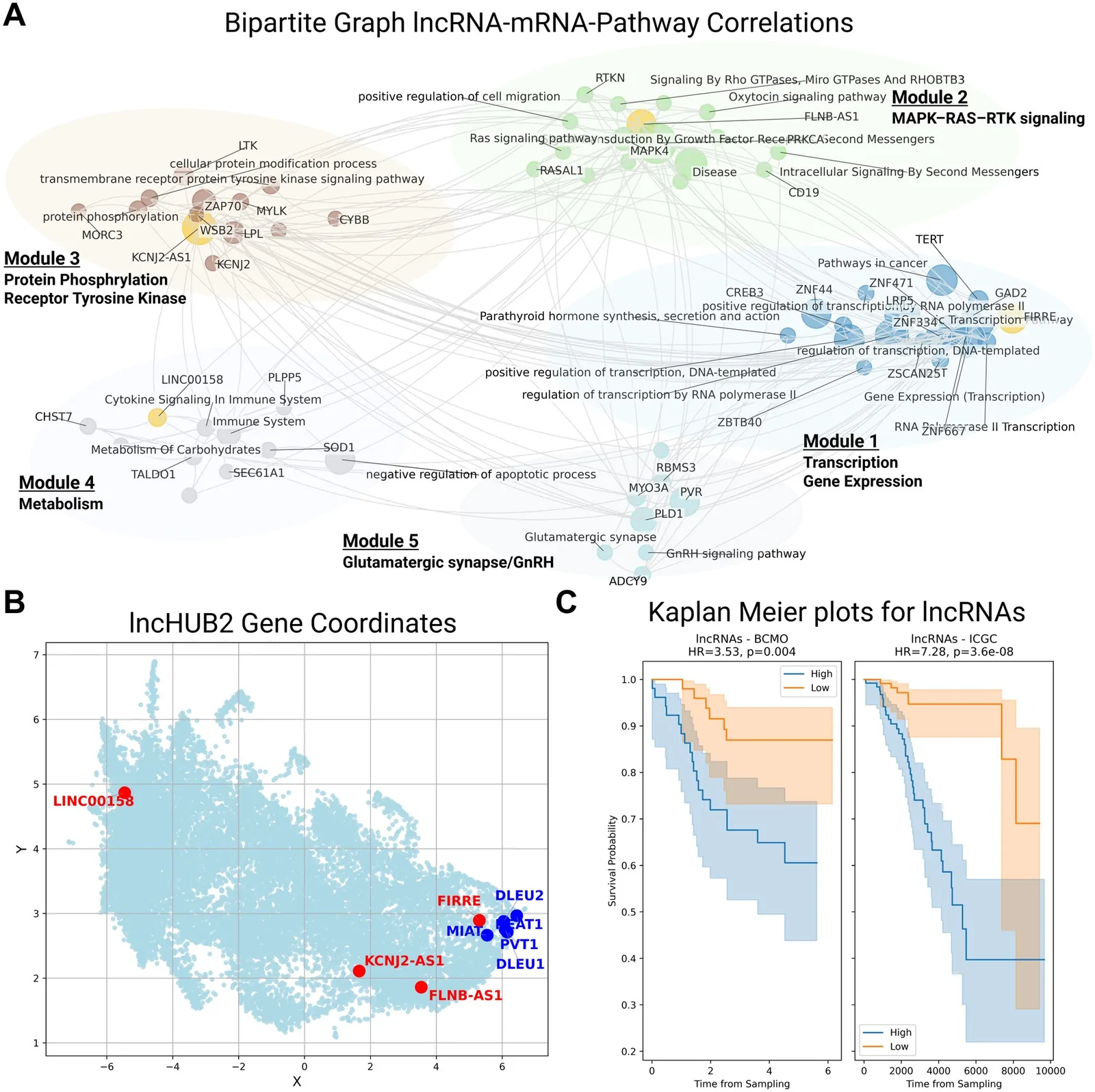
*Post hoc* analysis applied to the CLL case study. (A) Bipartite graph showing correlations among drivers and between drivers and pathways, and clustered by the community-Leiden algorithm. lncRNAs were coloured in yellow-orange. (B) UMAP visualization from lncHUB2 showing how close the lncRNAs identified in the current analysis (red) are to those lncRNAs previously reported in CLL studies (blue). (C) Kaplan–Meier survival plots for each dataset (ICGC and BCMO) using lncRNAs selected from the top SHAP values, incorporating survival status and lncRNA activity values. Created with BioRender.com.

The graph is organized into five modules, each reflecting a major biological process. Module 1 centers on transcriptional regulation, within which FIRRE appears as a subgraph (Supplementary Fig. 4A). Module 2 represents a MAPK-RAS-RTK signalling hub that governs proliferation, migration, and growth-factor responses, and includes FLNB-AS1 as a subgraph. Module 3 (Supplementary Fig. 4B) involves protein phosphorylation and receptor tyrosine kinase signaling, supporting cellular responses to external stimuli, with KCNJ2-AS1 embedded as a subgraph (Supplementary Fig. 4C). Module 4 combines immune and metabolic pathways, such as cytokine signaling and carbohydrate metabolism, and contains LINC00158 as a subgraph (Supplementary Fig. 4A). Finally, Module 5 integrates neuronal, hormonal, and gene-expression pathways, including glutamatergic synapse and GnRH signaling (Fig. 4A).

#### 3.2.8 Validation with ground-truth lncRNAs in CLL

Using the lncHUB2 pipeline and examining the UMAP projection, we observed that several lncRNAs from our analysis cluster closely with previously reported CLL-associated lncRNAs (Fig. 4B). In particular, FIRRE were found to be statistically closer to the known CLL-related lncRNAs, with observed average distances of 0.332 (Supplementary Table 1).

In contrast, other lncRNAs in our dataset showed associations with a broader range of cell types and tissues. This suggests that although not all lncRNAs are closely associated with the established CLL-associated cluster, those enriched in lymphoid tissues and lymphocyte-related cell types may still play biologically significant roles in CLL pathogenesis and warrant further investigation. Specifically, FIRRE and LINC00158 exhibited high expression in lymphoblastic and lymphoblastoid tissues. Moreover, FIRRE, LINC00158, and KCNJ2-AS1 showed elevated expression in hematopoietic and lymphoid-derived cell lines, including REH, HL60, Raji, and BJAB. These patterns highlight their potential relevance in lymphoid biology and suggest functional involvement in CLL-related cellular contexts (Supplementary Fig. 5).

**Figure 5:**
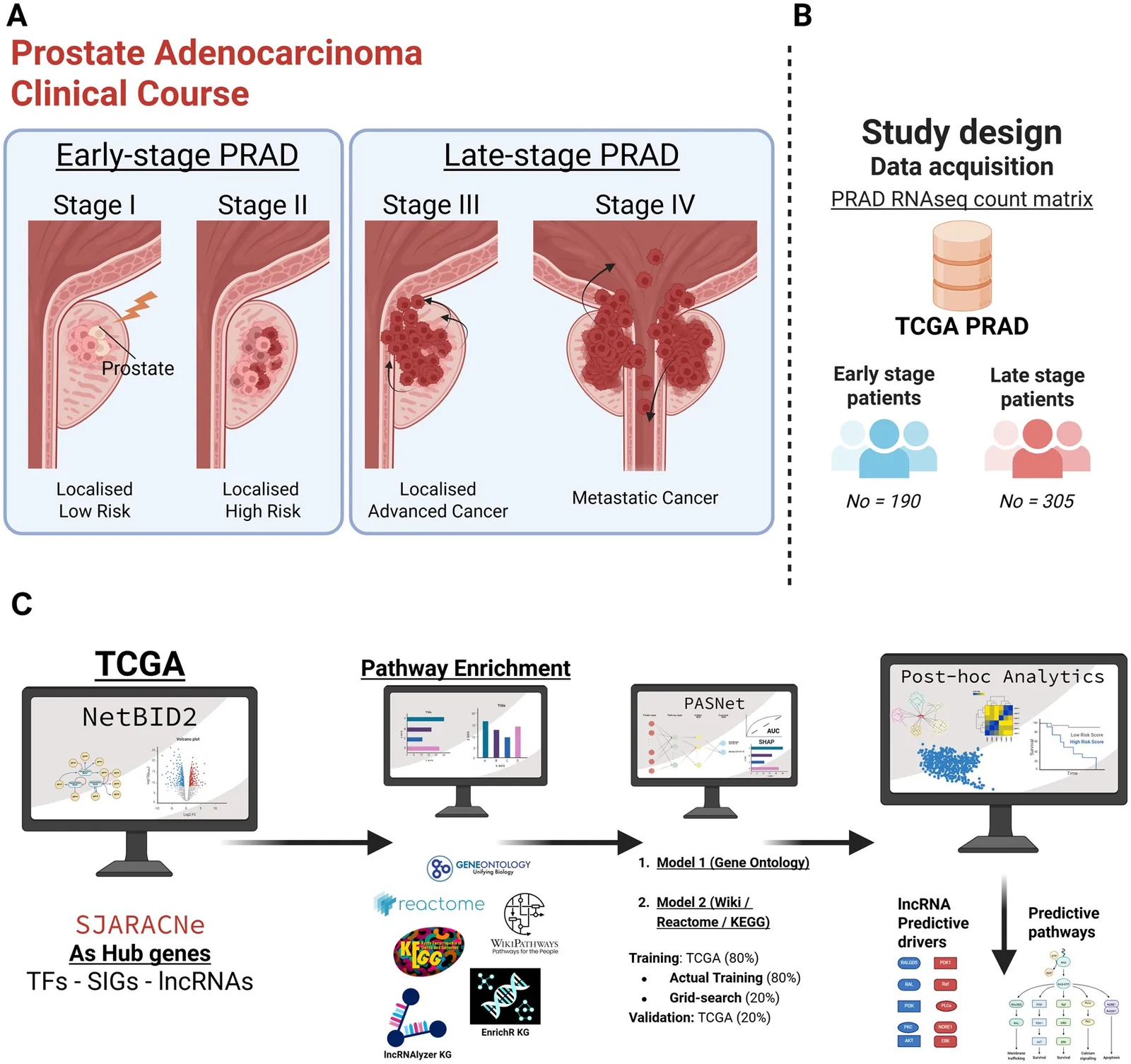
The lncAPNet framework applied to the PRAD case study. (A) A concise overview of PRAD progression, summarizing the key pathological and clinical features that characterize each stage of the disease. (B) Summarize the patients that stratified into early and late stages according to the metadata. (C) A schematic overview of the lncAPNet framework is shown for the PRAD case study. Created with BioRender.com.

#### 3.2.9 Prognostic value of discovered lncRNAs in CLL

Based on the most significant lncRNA drivers identified in the previous analysis, the samples were split into equal groups (50%–50%) by whether each lncRNA was more or less active, and their survival was evaluated using Kaplan–Meier plots. Survival status was retained, and *P* values and hazard ratios (HR) were calculated for each dataset based on activity values, following the NetBID2 approach for Kaplan–Meier plots using activity values. Notably, all selected lncRNAs appeared to be critical across both datasets (Supplementary Fig. 6). Moreover, when all lncRNAs were combined to assess their collective contribution to patient survival, the Kaplan–Meier analysis revealed significant associations in both datasets: BCMO (HR = 3.53, *P* = .004) and ICGC (HR = 7.69, *P* = 1.1 × 10^−8^) (Fig. 4C).

**Figure 6:**
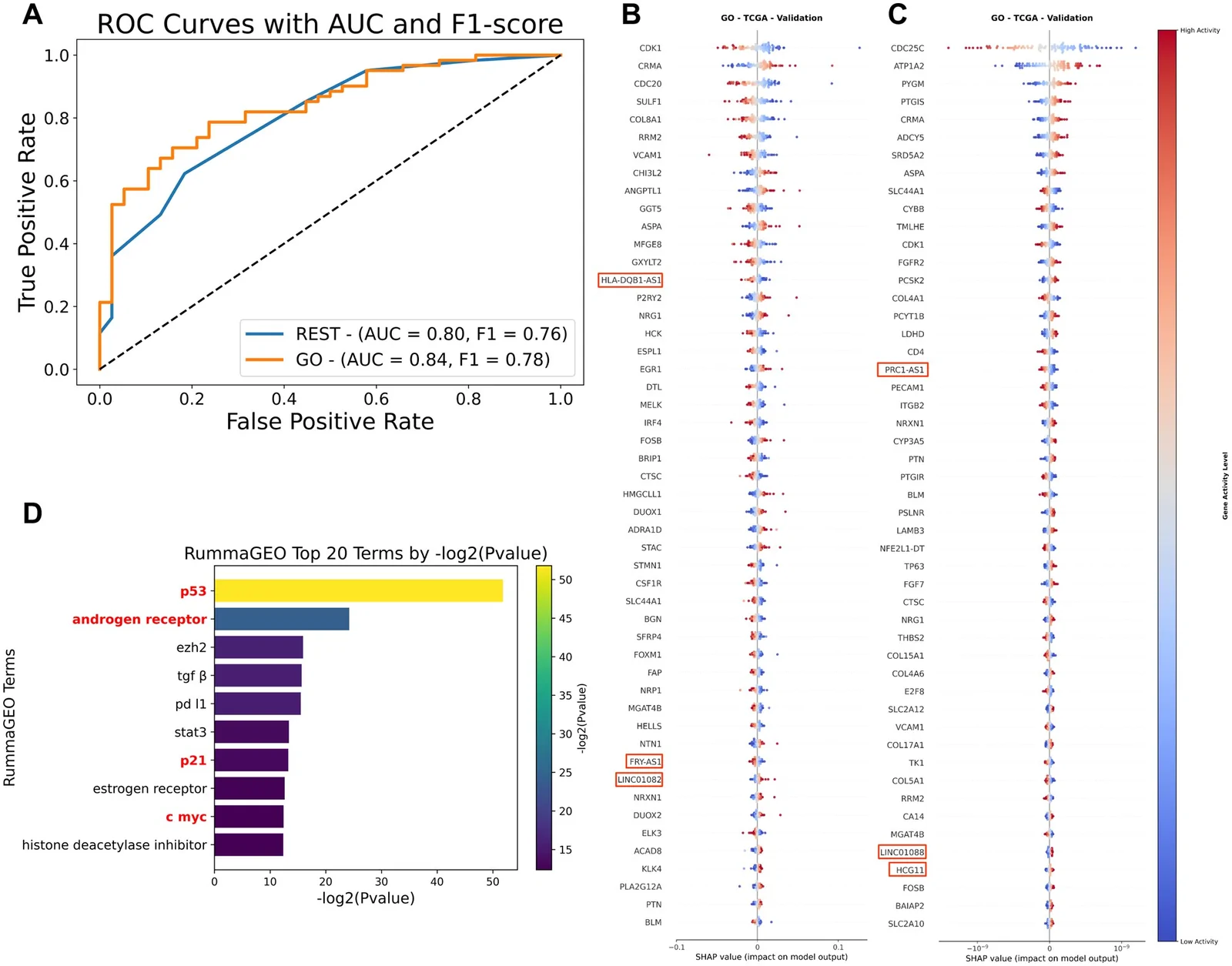
lncAPNet results in the PRAD case study. (A) ROC curves showing AUC and F1 scores for different lncAPNet configurations, including validation on the GO database and other databases. (B, C) Top 50 SHAP features displayed as SHAP plots for each comparison scenario: TCGA validation with the GO database (B) and with the remaining databases (KEGG, Reactome, and WikiPathways) (C). (D) Pathway enrichment analysis (RummaGEO) based on the top SHAP features from all analyses, with terms associated with PRAD highlighted in red on the *y*-axis. Created with BioRender.com.

### 3.3 Case study 2: Prostate adenocarcinoma—PRAD

#### 3.3.1 PRAD case study overview

The second scenario focused on PRAD, in which patients were stratified according to tumor stage, distinguishing early-stage (Stages I–II) from late-stage (Stage III–IV) disease to reflect increasing tumor burden, aggressiveness, and metastatic potential (Fig. 5A). Additional clinical manifestations such as the sum of individual Gleason Grades (primary and secondary patterns) were also included as criteria for stratifying patients. The N and M classifications serve as more specific indicators of disease progression, denoting lymphatic involvement and distant metastasis, respectively. Taken together, these variables contribute to determining the overall disease stage (Cheng *et al.* 2012). The objective was to identify lncRNAs associated with tumor progression and malignancy, thereby capturing molecular change underlying disease progression. Although comprehensive clinical data for survival analyses were not available in this cohort, lncAPNet was successfully applied to extract candidate lncRNA drivers correlated with cancer stage, providing insights for tumor progression.

#### 3.3.2 Data retrieval and appropriate format creation

PRAD bulk RNA-seq count matrix and metadata were retrieved from The Cancer Genome Atlas (TCGA) via the Genomic Data Commons (GDC) Data Portal, through the TCGA-PRAD project. The clinical characteristics of the samples were categorized into early (Stages I, II) and late (Stages III, IV) stages of cancer, indicating the malignancy of the cancer. Specifically, the number of early-stage cancer patients was Ne = 190, whereas the number of late-stage patients was Nl = 305. Finally, after filtering and transcript annotation, the total number of annotated transcripts was 33 403 (Fig. 5B).

##### 3.3.3 Activity analysis and identification of driver genes

The second case study focuses on the malignancy of PRAD, splitting the dataset into early stages of cancer (Stages I, II) and late stages of cancer (Stages III, IV).

Similar to the first case study scenario, we applied the SJARACNe algorithm for network reconstruction using its relevant gene lists (TFs, SIGs, lncRNAs), with the algorithm’s default parameters (number of epochs set to 100, consensus *P*-value threshold at 5 × 10^−8^).

The NetBID2 algorithm was applied again to the entire RNA-seq dataset, comparing the two groups as described above. Similarly to the previous case study scenario, we kept the drivers that have adj *P*-value < .05 and at least 30 target genes (as recommended by the NetBID2 pipeline for reliable drivers inference) from the SJARACNe algorithm. Notably, the significant drivers that were extracted from this filtering comprised 4331 [2006 hyperactive and 2325 hypoactive drivers]. Interestingly, only 2.609 of those drivers were noted as overt drivers, those that are important with the differential Expression method, whereas 1.722 of the mentioned drivers were counted as hidden, whose expression analysis was not statistically significant (Supplementary Fig. 7).

**Figure 7:**
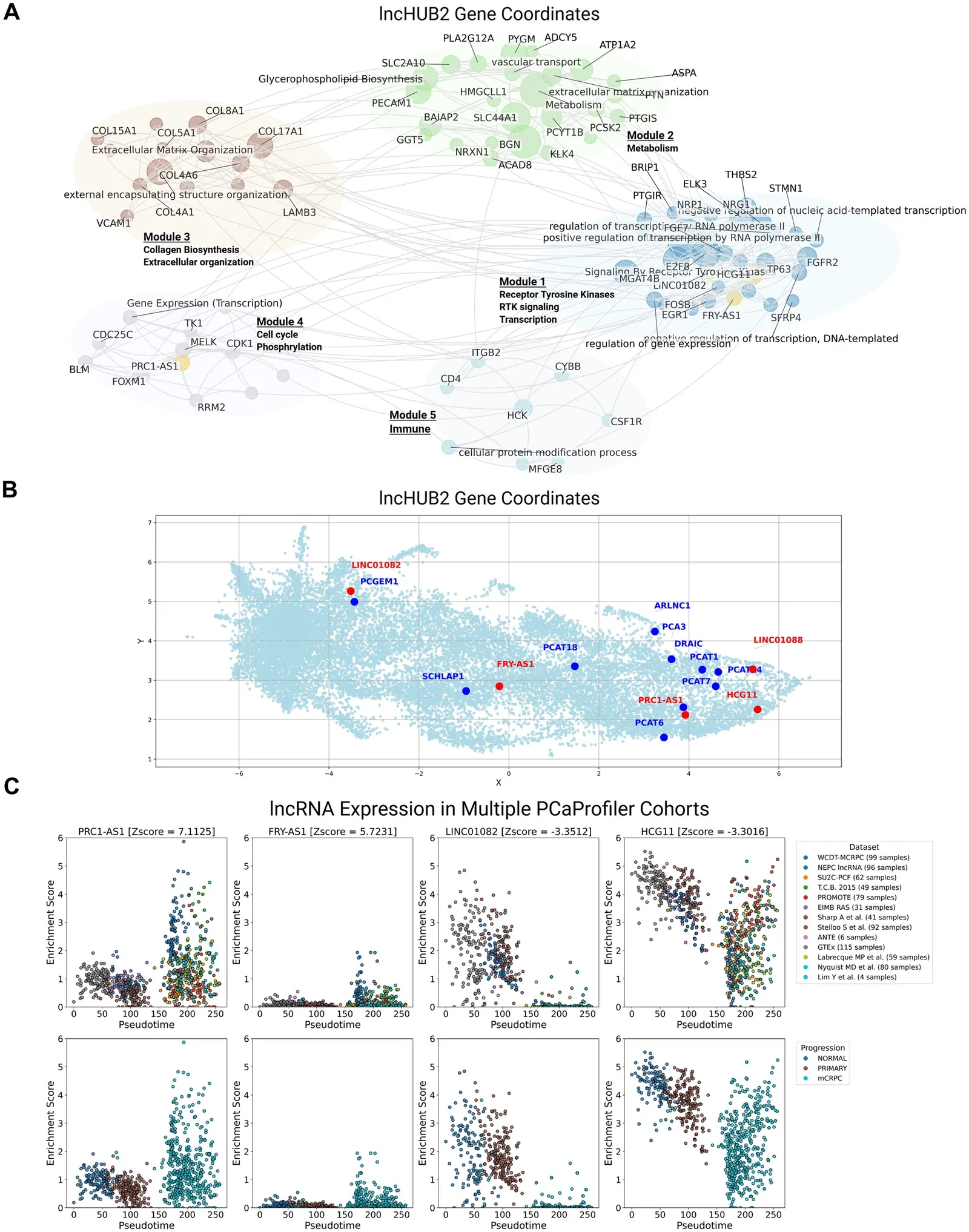
*Post hoc* analysis applied to the PRAD case study. (A) A bipartite graph illustrates the correlations among driver genes as well as between drivers and pathways, with communities identified using the Leiden clustering algorithm, lncRNAs are highlighted in yellow–orange for visual distinction. (B) A UMAP projection from lncHUB2 displays the spatial relationship between lncRNAs identified in the current analysis (red) and lncRNAs previously reported in prostate cancer studies (blue), showing their relative proximity in expression space. (C) Scatter plots derived from the PCaProfiler demonstrate, using independent datasets (upper panels), how the expression of the lncRNAs of interest changes along prostate cancer progression inferred through pseudotime analysis (lower panels). For each lncRNA, its corresponding *Z*-score from the differential activity analysis is also provided. Created with BioRender.com.

##### 3.3.4 Pathway enrichment analysis

In a total of 4331 significant drivers, we applied Pathway Enrichment analysis and set a cutoff of adjusted *P* values at .05. We extracted 140, 14, 92, and 0 pathways from GO, KEGG, Reactome, and Wikipathways, respectively.

Due to the large number of GO pathways, the analysis was separated into two distinct model training scenarios: (i) GO with 140 pathways, involving 1756 out of 4331 drivers, and (ii) with the rest of the databases, with 106 pathways, involving 1490 out of 4331 drivers (Supplementary Fig. 8).

**Figure 8:**
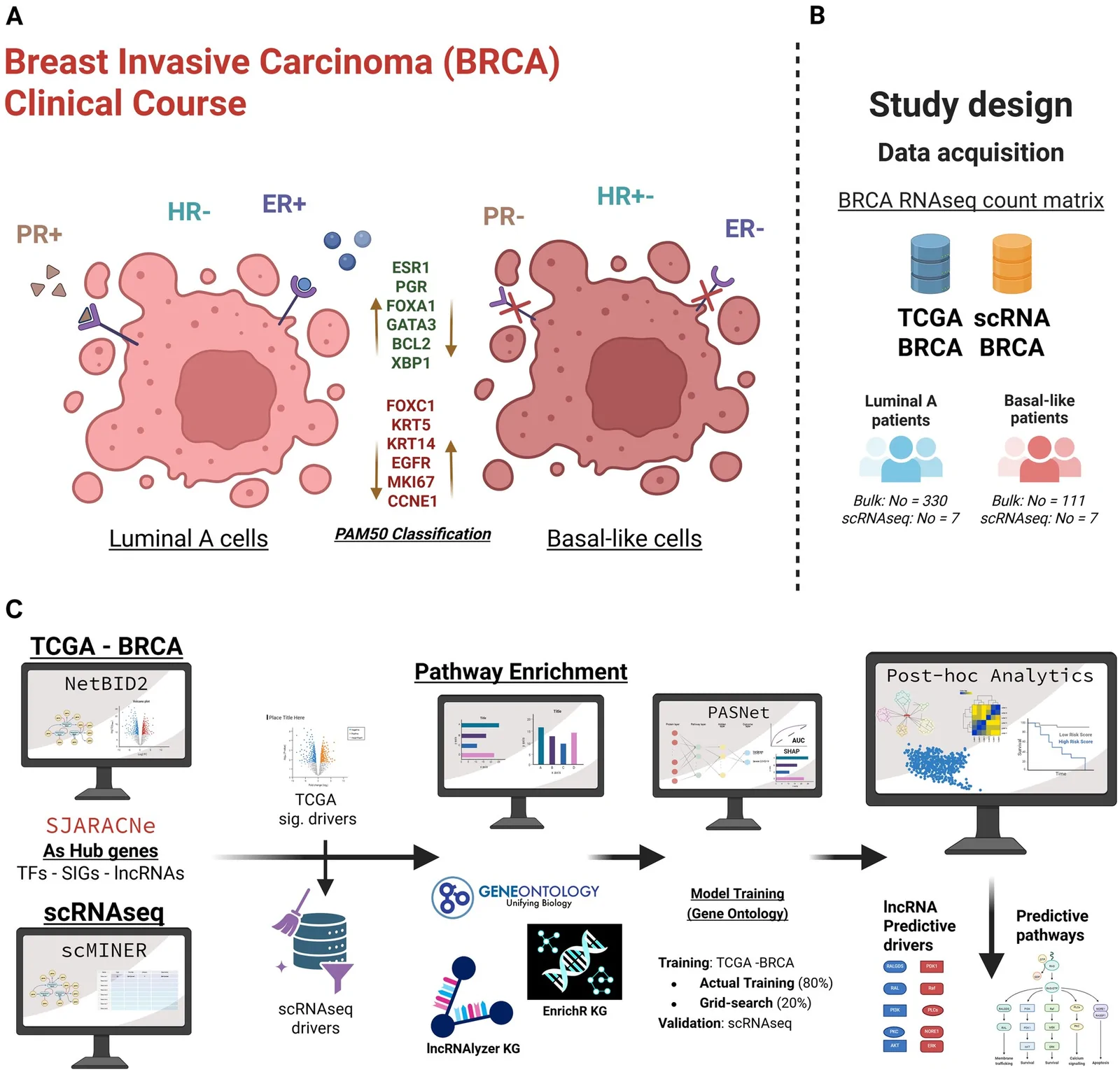
The lncAPNet framework applied to the BRCA case study. (A) Brief explanation of the differences between Luminal A and Basal-like BRCA cells, including their known clinical prognostic biomarkers based on, and (B) a summary of the BRCA patients in the dataset are stratified by Luminal A and Basal-like status. (C) Οverview of lncAPNet applied in the BRCA case-study scenario. Created with BioRender.com.

##### 3.3.5 Model training and validation

Similarly, for the CLL scenario, the dataset was first split 80%–20% into training and validation sets. The 80% training portion was then further divided into training and grid-search validation subsets using an 80%–20% split. For the grid search, the second training subset was used, and the grid-search validation subset was employed to determine the optimal Learning rate (LR) and L2 regularization parameters at 10 000 epochs, with drop-out rates [0.8, 0.7]. These optimized parameters were then applied to the final model training using the refined training and validation subsets.

For GO-based analysis, the TCGA training and TCGA validation datasets yielded an optimal learning rate (LR = 0.01), L2 regularization (L2 = 0.0003), and drop-out rates of 0.8 and 0.7. Subsequently, lncAPNet was trained and validated on the TCGA datasets, achieving robust performance metrics (AUC = 0.84, F1 score = 0.78) (Fig. 6A).

For the combined KEGG, Reactome, and WikiPathways analysis, the optimal hyperparameters were LR = 0.007 and L2 = 0.0003, with drop-out rates of 0.8 and 0.7, whereas validation on the TCGA datasets achieved an AUC of 0.79 and an F1 score of 0.76 (Fig. 6A).

##### 3.3.6 Identification of key lncRNA drivers

In this step, SHAP was employed to identify the most influential lncRNA features to the model’s predictions. For each model, the top 50 features ranked by SHAP values were extracted and combined into a unique list of significant features (87 drivers) (Fig. 6B and C). The SHAP threshold was increased from the top 20 to the top 50 values to capture more features. This compensates for validating the model solely on the split TCGA-PRAD dataset rather than an additional independent dataset. Notably, six lncRNAs were consistently ranked among the highest-scoring features: PRC1-AS1, FRY-AS1, LINC0182, HLA-DQB1-AS1, LINC01088, and HCG11. Enrichment analysis using RummaGEO indicated that these top-ranked drivers were linked to biological molecules implicated in the disease (Fig. 6D).

#### 3.3.7 Bipartite graphs driver-pathway correlations

Following the extraction of driver-pathway correlations from PASNet, we focused on the most meaningful associations by excluding those within the interquartile range (25th–75th percentile). Next, we combined GO results with additional pathway databases, including KEGG, Reactome, and WikiPathways, and implemented a filtering step to retain only pathways linked to at least three drivers and drivers involved in at least one pathway. Using this strategy, we pinpointed four lncRNAs [PRC1-AS1, FRY-AS1, LINC0182, and HCG11] that showed strong correlations with these pathways (Supplementary Fig. 9).

**Figure 9:**
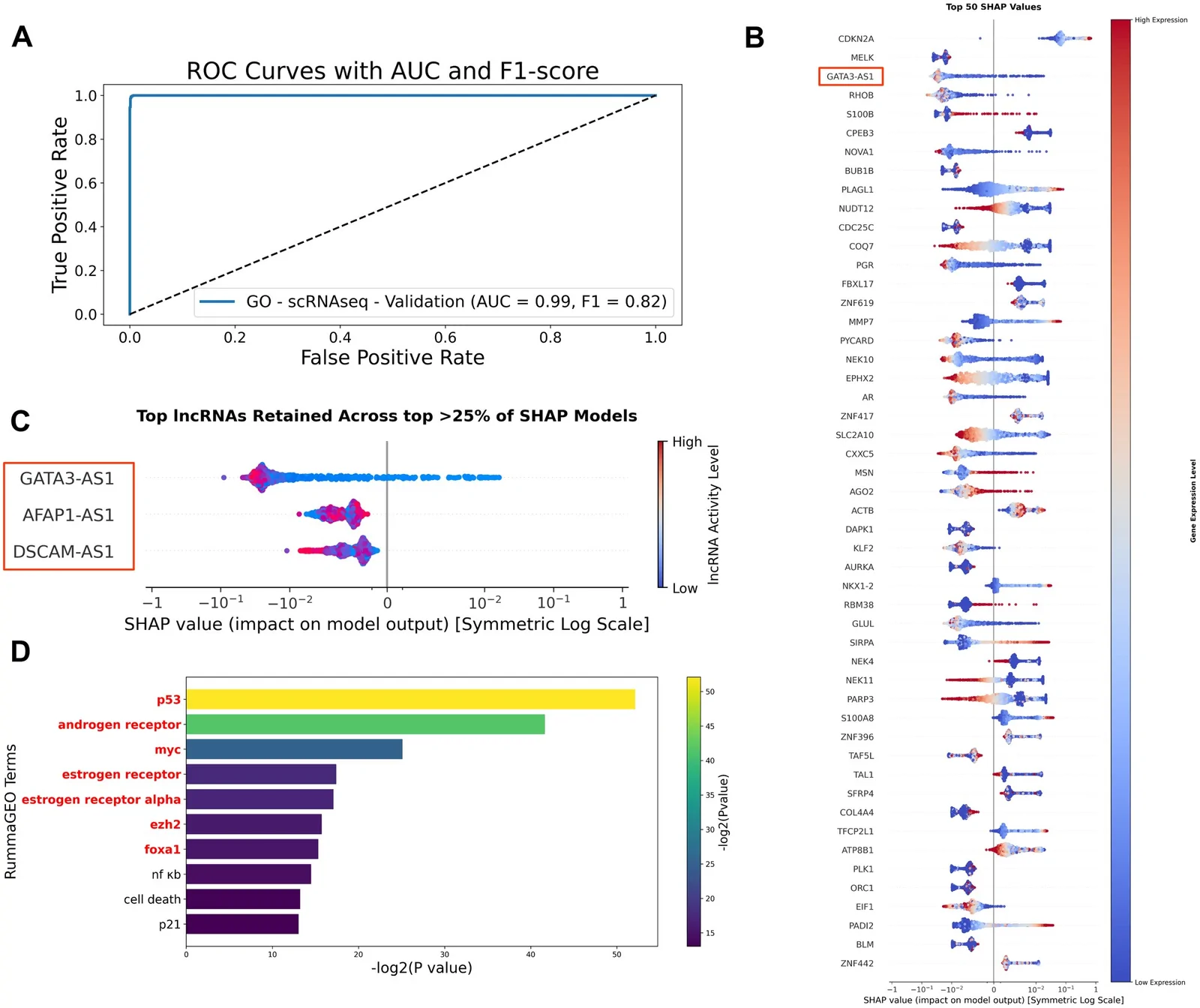
lncAPNet results in the BRCA case study: (A) ROC curves showing AUC and F1 scores for lncAPNet training model, by using GO for biological priors and scRNAseq for validation. (B) Top 50 SHAP explainable features displayed as SHAP plot from the trained model. (C) lncRNAs that exist in the top 25% of SHAP features from analysis. (D) Pathway enrichment analysis (RummaGEO) based on the top SHAP features from all analyses, with terms associated with BRCA highlighted in red on the *y*-axis. Created with BioRender.com.

The network organizes into distinct clusters that represent key biological processes. Module 1, which includes FRY-AS1, LINC0182, and HCG11, is enriched for transcriptional regulation and RTK signaling, featuring pathways such as RNA Polymerase II transcription, regulation of transcription, and signaling by receptor tyrosine kinases (Supplementary Fig. 10A). Module 2 centers on metabolic processes, encompassing glycerophospholipid biosynthesis, phospholipid metabolism, amino acid catabolism, and pathways related to the extracellular matrix and vascular transport. Module 3 highlights extracellular matrix and collagen organization, including collagen biosynthesis and modifying enzymes, collagen chain trimerization, and extracellular structure organization. Module 4, which contains PRC1-AS1 (Supplementary Fig. 10B), focuses on the cell cycle and phosphorylation, with pathways such as G1/S-specific transcription, gene expression, and protein phosphorylation. Finally, Module 5 involves immune signalling and protein modification (Fig. 7A).

**Figure 10:**
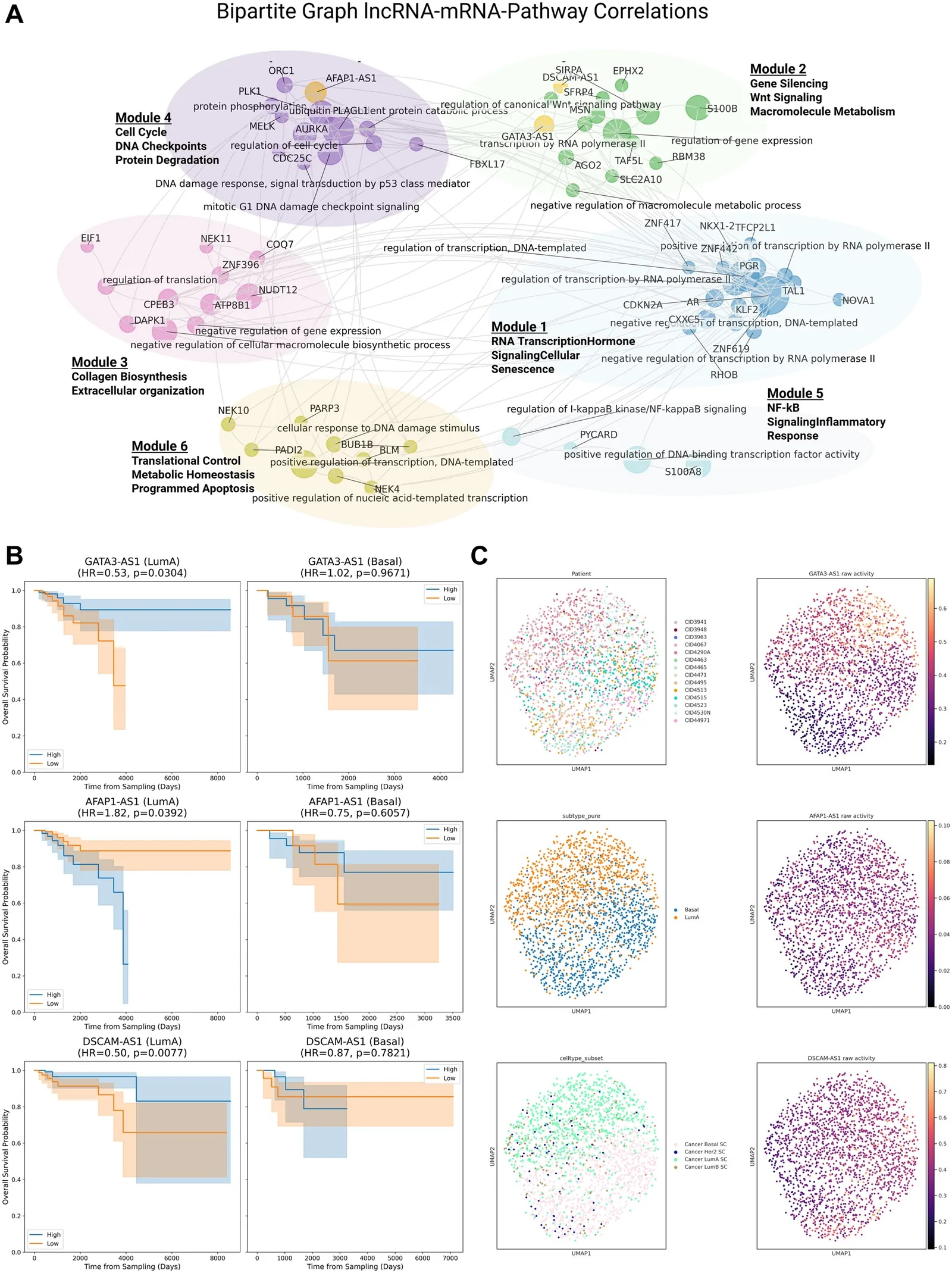
*Post hoc* analysis applied to the BRCA case study: (A) A bipartite graph illustrates correlations among driver genes and between drivers and pathways. The Leiden clustering algorithm identifies communities within this network. For visual distinction, lncRNAs are highlighted in yellow–orange. (B) Kaplan–Meier survival plots show outcomes for the target lncRNAs, with samples stratified into LumA and Basal subtypes. The analysis incorporates survival status and lncRNA activity values, adjusted for age and AJCC pathologic *N* stage covariates. (C) UMAP plots show cell-type characteristics (Samples, Status, and cell types) in scRNA-seq data. Additional plots display the activity levels of the target lncRNAs. Created with BioRender.com.

#### 3.3.8 Validation with ground-truth lncRNAs in PRAD

Using the lncHUB2 pipeline and examining the UMAP projection, we observed that several lncRNAs from our analysis cluster closely with previously reported Prostate cancer-associated lncRNAs (Fig. 7B). In particular, PRC1-AS1 and LINC01082 were found to be statistically closer to the known PRAD-related lncRNAs, with observed average distances of 0.200 and 0.284, respectively (Supplementary Table 2).

In contrast, other lncRNAs in our dataset exhibited associations with a broader range of cell types and tissues. This indicates that although not all lncRNAs are closely linked to the established prostate-associated cluster, PRC1-AS1 and LINC01082 are enriched in sperm and spermatozoa, whereas HCG11 is enriched in the urogenital reproductive system. Additionally, HCG11 correlates with prostate-related cell lines such as DU-145 and FRY-AS1, as well as with C4-2. LINC01082 is also detectable in PC-3 cells, though its expression is very low and likely negligible (Supplementary Fig. 11).

Pseudotime in the Prostate Cancer Atlas (PCaProfiler) represents an inferred molecular trajectory that orders samples from normal to advanced disease based on global gene expression patterns. It captures gradual transcriptional changes that occur during prostate cancer progression rather than reflecting actual chronological time. Enrichment scores, calculated using ssGSEA, quantify pathway or gene-signature activity within each sample. Along pseudotime, PRC1-AS1 and FRY-AS1 show increasing enrichment, indicating elevated activity in advanced stages, whereas HCG11 decreases and LINC01082 becomes nearly absent. These patterns align with differential activity *Z*-scores, with PRC1-AS1 and FRY-AS1 showing positive activity and HCG11 and LINC01082 showing negative activity. Together, these findings highlight stage-specific lncRNA dynamics and their potential roles in promoting prostate cancer progression (Fig. 7C).

### 3.4 Case study 3: Breast invasive carcinoma—BRCA

#### 3.4.1 Multi-omic BRCA case study overview

The final case study evaluated BRCA progression by integration of bulk and single-cell RNA sequencing data. Using cohorts from TCGA and the Breast Cancer Atlas, patients were stratified by transcriptomic cell states to contrast Luminal A tumors with aggressive, metastatic Basal-like phenotypes. Luminal A breast cancer is characterized by hormone receptor positivity (ER+, and PR+) and a slow-growing profile that responds well to endocrine therapy. Conversely, Basal-like breast cancer lacks these hormone receptors (ER−, and PR−) and overexpresses aggressive proliferation and structural markers, resulting in a faster-growing phenotype treated primarily with chemotherapy (Fig. 8A). Clinical evaluation relied on the categorical BRCA Subtype PAM50 classification variable (based on a 50-gene expression profile to predict tumor behavior and treatment response), which is rooted in the intrinsic molecular subtyping frameworks (Parker *et al.* 2009). This analysis aimed to pinpoint long non-coding RNAs (lncRNAs) that drive basal-like malignancies and to capture molecular transitions that drive advanced disease. Using the lncAPNet framework on long-term patient data from the TCGA-BRCA cohort, researchers identified potential driver lncRNAs associated with basal-like breast cancer progression. This provides a clearer look at how these tumors change over time.

#### 3.4.2 Data retrieval and appropriate format creation

BRCA bulk RNA-seq count matrices and corresponding clinical metadata were retrieved from TCGA via the Genomic Data Commons (GDC) Data Portal under the TCGA-BRCA project. Samples were stratified into *lumA* (*N* = 330) and *basal* (*N* = 111) cohorts based on the PAM50 molecular subtype classification. Following quality control filtering and transcript annotation, a final total of 32 122 annotated transcript IDs were retained. For the single-cell RNA-seq (scRNA-seq) dataset, data were obtained from the Broad Institute Single Cell Portal under the BRCA study repository. Cancer epithelial cells were exclusively retained for downstream analysis. To account for cellular heterogeneity within the initial pool of 20 samples, the relative percentages of cell types were quantified. Based on these proportions, the samples were classified into four intrinsic subtypes: LumA, LumB, HER+, and Basal. Downstream integration was then performed using the 7 predominantly Luminal A samples and 7 predominantly Basal-like samples (Supplementary Fig. 12). Finally, cellular downsampling was performed to yield a balanced dataset of 1000 Luminal A cells and 1000 Basal-like cells, encompassing a final feature space of 29 733 genes (Fig. 8B).

##### 3.4.3 Activity analysis and identification of driver genes

The final case study examines basal-like BRCA malignancy by partitioning each dataset into Luminal A and Basal subtypes. Consistent with the previous case studies, we applied the SJARACNe algorithm with default parameters (100 epochs and a consensus *P*-value threshold of 5 × 10^−8^) for network reconstruction using specific gene lists, including transcription factors, signaling molecules, and long non-coding RNAs. SJARACNe was executed independently for each dataset type using the count matrices.

The NetBID2 algorithm was applied to the bulk TCGA RNA-seq dataset. Adhering to the recommended NetBID2 pipeline for robust driver inference, we selected drivers with an adjusted *P*-value < .05 and at least 30 target genes from the SJARACNe network reconstruction. This filtering strategy identified 7538 significant drivers, comprising 3815 hyperactive and 3723 hypoactive. Within this cohort, 11 855 drivers were identified as overt (statistically significant through both differential activity and differential expression analysis), while 2940 were classified as hidden drivers, exhibiting no statistically significant changes in activity level (Supplementary Fig. 13). For the scRNA-seq dataset, driver activity was estimated using the scMINER algorithm in conjunction with SJARACNe. In this step, no statistical significance filtering was applied to the genes; only the activity matrix was used to align them with the statistically significant drivers from the TCGA dataset. Intersecting the gene lists between both modalities yielded a final subset of 4507 overlapping drivers retained across both datasets.

##### 3.4.4 Pathway enrichment analysis

For this case study, pathway enrichment analysis was restricted to the Gene Ontology (GO) database. Submitting the 4507 significant drivers to this analysis using an adjusted *P*-value cutoff of .05 yielded 92 enriched GO terms. To accommodate the extensive number of pathways, model training was partitioned into distinct scenarios, beginning with a model utilizing all 92 pathways, which encompassed 2291 of the 4507 drivers across both datasets.

##### 3.4.5 Model training and validation

Before implementing the PASNet algorithm, the dataset was partitioned into an 80% training set and a 20% validation-tuning set. Hyperparameter optimization was then performed using grid search on a dedicated training subset and a validation subset to identify the optimal learning rate (LR) and L2 regularization parameters over 10 000 epochs, with drop-out rates [0.8 and 0.7]. These optimized hyperparameters were subsequently used to train the final model on the refined training and validation subsets.

For the GO-based analysis, a grid search on the TCGA training and validation subsets yielded an optimal LR of 0.007 and an L2 regularization value of 0.0003. Using these parameters, the lncAPNet model was trained on the TCGA training cohort and validated on the scRNA-seq dataset. It achieved robust performance, with an AUC of 0.99 and an F1 score of 0.82 (Fig. 9A). This outstanding outcome reflects true generalizability rather than overfitting. This robustness is demonstrated by successful evaluation across entirely independent bulk and single-cell cohorts by using the activity values. By utilizing separate driver activity matrices derived from NetBID2 and scMINER instead of raw expression data, the model captures stable, conserved biological signals. This approach successfully filters out dataset-specific noise. Consequently, the high accuracy achieved across these distinct platforms confirms the cross-dataset validity of our predictive model.

##### 3.4.6 Identification of key lncRNA drivers

Subsequently, SHAP analysis was employed to identify the long non-coding RNA (lncRNA) features driving model predictions. The top 50 features were extracted based on their mean absolute SHAP values (Fig. 9B; Supplementary Fig. 14). Expanding this selection to the top 25% of features by SHAP value revealed two additional significant lncRNAs: AFAP1-AS1 and DSCAM-AS1 (Fig. 9C; Supplementary Fig. 14). While the top 50 list captured only GATA3-AS1, this broader threshold successfully isolated all three key features. Functional enrichment analysis using RummaGEO into top 50 SHAP features indicated that these top-ranked drivers were associated with biological molecules heavily implicated in BRCA pathogenesis (Fig. 9D).

#### 3.4.7 Bipartite graphs driver-pathway correlations

Based on the bipartite graphs and the architectural analysis of the provided gene clusters, each distinct regulatory module is driven by a specific combination of signalling pathways and long non-coding RNAs (lncRNAs) (Fig 10A). Module 1 (RNA Transcription, Hormone Signalling, Cellular Senescence) is characterized by the fine-tuning of the RNA polymerase II transcription pathway to modulate DNA-templated synthesis. Module 2 (Gene Silencing, Wnt Signalling, Macromolecule Metabolism) serves as a major metabolic hub driving gene expression regulation and the canonical Wnt signaling pathway, which is uniquely co-regulated by the lncRNAs DSCAM-AS1 and GATA3-AS1 (Supplementary Fig. 15A). Module 3 (Collagen Biosynthesis, Extracellular Organization) orchestrates post-transcriptional control by modulating the translation pathway and downregulating cellular macromolecule biosynthetic operations. Module 4 (Cell Cycle, DNA Checkpoints, Protein Degradation) governs genomic integrity and proliferation, mapping directly to the regulation of the cell cycle, protein phosphorylation, and the p53-mediated mitotic G1 DNA damage checkpoint while featuring the lncRNA AFAP1-AS1 (Supplementary Fig. 15B). Module 5 (NF-kB Signaling, Inflammatory Response) operates as an immune and stress-response axis that actively mediates the I-kappaB kinase/NF-kappaB signaling pathway. Finally, Module 6 (Translational Control, Metabolic Homeostasis, Programmed Apoptosis) is highly specialized for genomic maintenance, coordinating the cellular response to DNA damage stimulus alongside positive regulation of nucleic acid-templated transcriptions.

Clinical outcome analysis within the TCGA dataset demonstrates that the elevated activity of the three network-associated lncRNAs (GATA3-AS1, AFAP1-AS1, and DSCAM-AS1) significantly correlates with overall survival in Luminal A (LumA) BRCA patients, with multivariate Cox regression confirming this independent prognostic value after adjusting for age and AJCC pathologic *N* stage (Fig. 10B). Furthermore, evaluation of single-cell RNA-sequencing (scRNA-seq) data reveals that GATA3-AS1 exhibits markedly higher activity in LumA malignant epithelial cells compared to the basal-like subtype, underscoring its subtype-specific regulatory role. In contrast, the remaining lncRNAs, AFAP1-AS1 and DSCAM-AS1, do not display differences in activity between these BRCA subtypes (Fig. 10C).

### 3.5 Benchmarking

#### 3.5.1 Ablation study using the PASNet algorithm in expression values

To validate our methodology, we performed an ablation study using bulk RNA-sequencing (RNA-seq) expression data from The Cancer Genome Atlas (TCGA) alongside a single-cell RNA-seq (scRNA-seq) validation expression dataset, mapping both to Gene Ontology (GO) terms within the PASNet framework to extract biologically explainable features.

For this ablation analysis, we first filtered the TCGA count matrix to retain 11 756 statistically significant differentially expressed genes (DEGs), of which 8026 genes overlapped with the independent scRNA-seq validation dataset. Subsequent functional filtering restricted the feature space to significantly enriched Gene Ontology (GO) terms (*P* < .05), yielding a final input network composed of 5358 unique genes mapped across 498 GO pathways.

This filtered dataset was subsequently partitioned into training (80%) and validation (20%) cohorts. Hyperparameter tuning via grid search determined that the optimal network configuration utilized a learning rate of 0.0005, an L2 regularization weight of 0.0005, and dropout rates of 0.8 and 0.7 for successive layers. Under these optimized parameters, PASNet was trained on the TCGA bulk expression matrix integrated with GO pathways, while the independent scRNA-seq count matrix served as the final validation cohort.

#### 3.5.2 Benchmarking with other algorithms

To benchmark our framework against alternative linear and non-linear machine learning architectures, we trained and evaluated Random Forest (RF), XGBoost (XGB), Support Vector Machine (SVM), and Logistic Regression models (GLR) on the identical bulk RNA-seq training and scRNA-seq validation cohorts.

Performance was comprehensively evaluated using classification metrics (AUC and F1-score) alongside biological interpretability metrics, quantified via functional enrichment analyses across the Gene Ontology (GO), Reactome, KEGG, and WikiPathways databases, as well as protein-protein interaction (PPI) network densities from STRINGdb. Although we used the Gene Ontology as a pathway enrichment database and our dataset was trained on it, which could probably introduce bias, the pathways from the other databases still yielded more biologically meaningful results.

To assess explainability, we extracted the top 50 features from each model based on SHAP values. Our proposed lncAPNet framework not only demonstrated superior predictive performance in terms of AUC and F1-score, but also exhibited markedly higher biological explainability. Specifically, the SHAP-derived feature sets from lncAPNet yielded significantly tighter functional pathway enrichment scores and a higher density of documented gene-gene correlation links within STRINGdb compared to the baseline models (Table 1).

**Table 1:** Summary of the benchmarking metrics.

Evaluation metrics	lncAPNet	Ablation study	RF	XGB	SVM	GLR
AUC	0.99	0.99	0.49	0.5	0.99	0.99
F1score	0.82	0.89	0.66	0.66	0.66	0.95
Enrichment (*P*-value < .05)						
GO	229	34	28	18	18	6
KEGG	3	4	1	0	0	0
Reactome	22	5	1	5	7	14
Wikipathway	18	3	14	0	3	13
STRINGdb connections						
Edges	57	11	33	10	13	19

## 4 Discussion

The therapeutic potential of lncRNAs has garnered significant attention in recent years, with several candidates advancing toward clinical evaluation. lncRNA expression can be modulated through multiple approaches, including siRNAs, antisense oligonucleotides, CRISPR-Cas9, and small molecule inhibitors (Tamblin-Hopper *et al.* 2024), with several preclinical programs demonstrating promising results. Notably, HAYA Therapeutics is advancing HTX-001, which targets the lncRNA WISPER, a master regulator of cardiac fibrosis, toward clinical trials for heart failure treatment. The tissue and cell-type specificity of lncRNAs makes them particularly attractive therapeutic targets, as their restricted spatial and temporal expression patterns enable lineage-specific gene therapy with potentially reduced off-target effects (Tamblin-Hopper *et al.* 2024). Well-characterized lncRNAs such as MALAT1 (Puri *et al*. 2025), HOTAIR (Zhou *et al.* 2015), and ANRIL (Yin *et al.* 2021), which have been implicated in cancer progression, cardiovascular disease, and inflammatory conditions, exemplify the breadth of diseases amenable to lncRNA-targeted interventions.

Our pipeline’s ability to systematically identify lncRNA–mRNA–pathway interactions provides a framework for prioritizing candidate lncRNAs based on their regulatory roles in disease-relevant pathways, potentially accelerating the discovery of novel therapeutic targets. A key function of the entire workflow is to enhance the signal of lncRNAs, treating them as hub molecules across various biological systems and aligning their values with protein-coding transcripts. While the original NetBID2 and scMINER workflows (Dong *et al.* 2023, Pan *et al.* 2025) only recognized transcription factors (TFs) and signaling genes (SIGs), the lncAPNet workflow also incorporates lncRNAs and calculates their activity values to boost their signal in the system. Also, while lncRNAlyzr (Evangelista *et al.* 2025) represents a valuable resource for exploring lncRNA-mRNA relationships through its knowledge graph structure, its current implementation remains relatively static, limiting its utility for data-driven biological discovery and clinical decision-making. To address this limitation, we propose integrating deep learning approaches that can leverage the knowledge graph architecture while maintaining biological interpretability. By enabling deep learning models to access and learn from the knowledge graph’s structured relationships, we can facilitate biologically informed decisions, such as supervised patient stratification, in which the model not only performs clinical classifications but also reveals which specific lncRNA-mRNA connections drive its predictions. This integration forms the basis of lncAPNet, a comprehensive framework that synergistically combines knowledge-graph relational connections, co-expression networks derived from SJARACNe, deep-learning classification models, explainable AI via SHAP (SHapley Additive exPlanations) values, and network analytics. This multi-layered approach enables researchers to move beyond simple database queries toward a mechanistic understanding, identifying the key lncRNA-mRNA regulatory axes that underlie disease phenotypes and potentially guiding the selection of lncRNA therapeutic targets based on their functional relevance in patient subgroups.

The cornerstone of lncAPNet is an activity inference strategy that relies on both activity values and biological priors (knowledge), with a specific focus on lncRNAs. As emphasized in the original APNet (Gavriilidis *et al.* 2025), activity values provide two key advantages: (i) better reflect regulatory dependencies between lncRNAs and protein-coding molecular entities, and (ii) enable the extraction of biologically meaningful lncRNA driver-pathway relationships. These properties are particularly crucial for lncRNAs, whose functions are often mediated through complex, nonlinear interactions with mRNAs.

In the CLL case study, the analysis identified a set of significantly dysregulated signalling and survival pathways correlated with the unmutated IGHV phenotype. These include heightened activation of the B-cell receptor (BCR) (Chen L *et al.* 2008, Chen J *et al.* 2024) and downstream tyrosine kinase-PI3K/AKT/MAPK/Ras pathways (Murali *et al.* 2021), enhanced cytokine and immune regulatory signalling (e.g. IL-4, CD40) (Coscia *et al.* 2010), increased engagement of the anti-apoptotic BCL2/NF-κB survival program (Majid *et al.* 2008), and greater responsiveness to microenvironmental and migration cues. Notably, genes such as LPL, ZAP70, PRKCA, and CD19 (Maloum *et al.* 2009, Puente *et al.* 2015, Chen J *et al.* 2024) were identified as critical molecules of disease progression in the unmutated IGHV status.

Among the significant lncRNAs, FIRRE emerged as a potential key driver across the analysis, demonstrating a strong association with both survival status and CLL-related lncRNA networks. According to existing literature, FIRRE, through the interaction with PTBP1, which activates the Wnt/β-catenin pathway, contributes to stabilizing BCR signalling in DLBCL, thereby promoting B cell proliferation and malignant progression (Liu Q *et al.* 2022).

In late-stage PRAD case studies, results show that correlated pathways and genes, including dysregulated receptor tyrosine kinase signalling driven by FGFR2, FGF7, and NRP1, activate PI3K/AKT (Wang *et al.* 2024) and MAPK pathways (Shen *et al.* 2021) to sustain proliferation, while CDK1, CDC25C, and TK1 promote cell cycle progression and genomic stability (Xie H *et al.* 2022). ECM remodelling and collagen biosynthesis (Stewart *et al*. 2004), mediated by COL4A1, COL5A1, COL8A1, COL15A1, and THBS2, enhance invasion and angiogenesis, complemented by transcriptional regulators FOXM1 (Liu Y *et al.* 2017), MELK/E2F8 (Lee *et al.* 2023), and FOSB (Barrett *et al*. 2017), which drive oncogenic programs. Secreted and adhesion molecules such as KLK4 (Klokk *et al.* 2007), PTN (Liu S *et al.* 2021), VCAM1 (Chen C *et al.* 2015), and CSF1R (Escamilla *et al.* 2015) further support angiogenesis and immune evasion, collectively promoting metastasis and tumor aggressiveness.

Notably, the lncRNAs PRC1-AS1, HCG11, LINC01082, and FRY-AS1 were identified as significant findings, potentially acting as key regulatory molecules within these oncogenic networks. Notably, reduced expression of the lncRNA HCG11 has been associated with the promotion of prostate cancer progression (Zhang Y *et al.* 2016) and has also been reported to inhibit the phosphoinositide 3-kinase/protein kinase B (PI3K/AKT) signalling pathway, thereby suppressing prostate cancer progression (Wang *et al.* 2019). Moreover, LINC01082 has been proposed as a potential prognostic biomarker, as it appears to play a critical role in patient survival (Zhang T *et al.* 2022). Consistently, downregulation of LINC01082 promoted 22RV1 cell migration, suggesting that LINC01082 may suppress prostate cancer cell motility by upregulating or activating DUSP2. These findings indicate that LINC01082 exerts its tumour-suppressive effects, at least in part, through the DUSP2-mediated pathway, potentially linked to the FOXA1-associated ceRNA network (Yang *et al.* 2023).

The distinct clinical behaviors of Luminal A (LumA) and Basal-like breast cancers are driven by specific mRNA expression profiles that regulate hormone signalling and cell division (Gaona-Romero *et al.* 2025). In Luminal A tumors, the highly expressed mRNAs PGR (progesterone receptor), AR (androgen receptor), and the long non-coding RNA GATA3-AS1 act as definitive biomarkers of a well-differentiated, hormone-receptor-positive phenotype tied to active estrogen signalling networks and favorable patient outcomes (Jacquemier *et al.* 2009, Contreras-Espinosa *et al.* 2021). Conversely, aggressive Basal-like breast cancers are characterized by the coordinated upregulation of proliferation-associated mRNAs, including MELK, AURKA, PLK1, and CDC25C. This mitotic mRNA expression cascade drives rapid cell cycle progression, genetic instability, and high-grade tumor proliferation, marking the aggressive, baseline profile that typically responds poorly to traditional endocrine therapies (Wang *et al.* 2014, Ingebriktsen *et al.* 2024).

While coding mRNAs act as the direct functional drivers of these breast cancer subtypes, long non-coding RNAs (lncRNAs) provide a critical layer of epigenetic and transcriptional regulation that reinforces these distinct phenotypes. Within Luminal A (LumA) tumors, the highly expressed lncRNAs GATA3-AS1 and DSCAM-AS1 function as lineage-specific markers of the hormone-receptor-positive phenotype, and are validated that play a critical role in Luminal A breast cancer cells (Sun *et al.* 2018, Contreras-Espinosa *et al.* 2021). These non-coding transcripts are directly tied to active estrogen receptor alpha ER + signaling networks, promoting a well-differentiated state and predicting more favorable patient outcomes while remaining largely silent in hormone-negative strains (Sun *et al.* 2018, Contreras-Espinosa *et al.* 2021). Conversely, aggressive Basal-like breast cancers are uniquely characterized by the significant upregulation of the oncogenic lncRNA AFAP1-AS1. This non-coding transcript is highly enriched in basal-like tumors, where it acts as a primary coordinator of hypoxia-activated angiogenesis, rapid cell proliferation, and metastatic progression, contributing heavily to the high-grade malignancy and chemotherapy-resistant nature characteristic of the basal phenotype (Rodrigues De Bastos and Nagai 2020).

## 5 Conclusions and future work

Despite its advantages, lncAPNet has several limitations in explaining biological mechanisms. First, the reliability of prior knowledge depends heavily on the comprehensiveness of current lncRNA databases, which remain less mature than those available for protein-coding genes. Second, functional interpretation of predicted lncRNA drivers must be approached with caution, as many lncRNAs are highly tissue- and context-specific. Third, co-expression does not necessarily imply direct interactions with their protein-coding targets. Rather, it provides a broader view of the pathways they participate in. Finally, a notable limitation of this framework is its inherent complexity, as a manual step-by-step execution could easily overwhelm the user. To overcome this challenge and streamline the process, we automated the entire workflow creating a Nextflow environment. This technological integration transforms a complex pipeline into an accessible tool, ensuring seamless portability, high reproducibility, and effortless scaling across diverse computing environments. Furthermore, we developed user-friendly Colab notebooks to provide *post hoc* explainability, significantly flattening the learning curve and enhancing workflow interpretability.

Looking forward, lncAPNet can be generalized across multiple cancer-disease settings, particularly where lncRNAs serve as biomarkers or therapeutic targets. Its activity-based integration of lncRNAs into DL frameworks offers a modular and expandable strategy. Indeed, this framework now incorporates single-cell multi-omics to capture cellular heterogeneity. Future iterations could further integrate spatial multi-omics to resolve spatial regulation, while *in vitro* and *in vivo* validation remain essential to establish translational value.

Overall, lncAPNet represents a conceptual and technical advance in interpretable deep learning for systems biology. By positioning lncRNAs as hub regulators within the activity-driven framework of APNet, it bridges the coding and non-coding layers of regulation, thereby improving both predictive power and mechanistic insight.

## Supplementary Material

vbag204_Supplementary_Data

## Data Availability

The lncAPNet R and Python scripts of the workflow of this manuscript can be found at: https://github.com/BiodataAnalysisGroup/lncAPNet. The RNA-seq count matrices and their relevant metadata that were used in this study can be accessed in the following links: ICGC ARGO [CLLE‑ES]: https://platform.icgc-argo.org/. BloodCancerMultiOmics2017 R package: https://bioconductor.org/packages/release/data/experiment/html/BloodCancerMultiOmics2017.html. TCGA PRAD: https://portal.gdc.cancer.gov/projects/TCGA-PRAD. TCGA BRCA: https://portal.gdc.cancer.gov/projects/TCGA-BRCA. scRNAseq Atlas: https://singlecell.broadinstitute.org/single_cell/study/SCP1039/a-single-cell-and-spatially-resolved-atlas-of-human-breast-cancers. All ExpressionSet formats and results of the above analyses are also included in Zenodo: https://doi.org/10.5281/zenodo.20637947. DOME Registry: https://identifiers.org/dome: xpjtohmv78.
