# Supplementary material for: lncAPNet enables the deciphering of lncRNA–mRNA connections in patient transcriptomic data
Source: Bioinform Adv. 2026 Jul 21;6(1):vbag204. doi: 10.1093/bioadv/vbag204 (PMC13485353; doi:10.1093/bioadv/vbag204)
Supplement: Supplementary file 1 [file vbag204_supplementary_data.zip › Supplementary_Materials.docx]

# Supplementary Figures

**Supplementary Figure 1.** Venn diagrams showing the overlapping positive and negative drivers across studies.

**Supplementary Figure 2.** Number of significant pathways identified in each database through enrichment analysis for the CLL case study. Bars represent the pathways, and the numbers above each bar indicate the number of associated drivers in each set.

**Supplementary Figure 3.** Heatmap depicting combined weight correlations between pathways (connected to at least three driver genes) from both models, along with top SHAP values from each model that are connected to at least one pathway for the CLL case study.

**Supplementary Figure 4.** **Submodule network representation of lncRNAs key driver hubs for CLL case study.** Functional interaction networks for four identified driver submodules: (A) LINC00158 Hub Module, (B) FLNB-AS1 Hub Module, (C) KCNJ2-AS1 Hub Module, and (D) FIRRE Hub Module. Node colors correspond to distinct functional categories and molecular classes: non-coding RNAs (NC yellow), signalling molecules (SIG blue), transcription factors (TF red), and biological pathways or functional terms (Pathway green). Edges represent documented regulatory interactions and functional associations within each hub environment.

**Supplementary Figure 5.** Box plots showing lncRNA expression across various tissues and cell types for the CLL case study scenario. For the CLL case study, the top 10 most highly expressed tissues (A) and cell types (B) for each lncRNA were extracted from ARCH4.

**Supplementary Figure 6.** Kaplan–Meier plots for each significant lncRNA identified in the CLL analysis. Survival curves are shown for samples with high (blue) versus low (orange) lncRNA activity. Plots are presented for (A) the BCMO dataset and (B) the ICGC dataset.

**Supplementary Figure 7. Distribution of Hyper-active and Hypo-active significant drivers in PRAD data.** The drivers were separated by activity state (Hyper/Hypo), driver class (Overt vs Hidden), and type [TF (blue), SIG (orange), and NC (green)]. Each bar represents the number of drivers in the corresponding category, with stacked colors indicating driver type.

**Supplementary Figure 8.** Number of significant pathways identified in each database through enrichment analysis for the PRAD case study. Bars represent the pathways, and the numbers above each bar indicate the number of associated drivers in each set.

**Supplementary Figure 9.** Heatmap depicting combined weight correlations between pathways (connected to at least three driver genes) from both models, along with top SHAP values from each model that are connected to at least one pathway, for the PRAD case study.

**Supplementary Figure 10.** **Submodule network representation of lncRNAs key driver hubs for BRCA case study.** This figure represents (A) PRC1-AS1 Co-Hub Module and (B) FRY-AS1/HCG11/LINC01082 Hub Module. Nodes represent functional categories [non-coding RNAs (yellow), signalling molecules (blue), transcription factors (red), and pathways (green)] with edges indicating regulatory interactions.

**Supplementary Figure 11.** Box plots showing lncRNA expression across various tissues and cell types for the PRAD case study scenario. For the PRAD case study, the top 10 most highly expressed tissues (A) and cell types (B) for each lncRNA were extracted from ARCH4.

**Supplementary Figure 12: Distribution and relative proportion of cell types per patient sample (BRCA).** Stacked bar plot showing the cellular composition of cancer epithelial cells across 20 BRCA patient samples (Patient ID). Samples are ordered by predominant cellular composition and grouped into the four intrinsic subtypes: Basal (red), Her2 (light blue), LumA (dark blue), and LumB (chartreuse/yellow). Patient labels on the horizontal axis are color-coded according to the final subtype classification of each sample.

**Supplementary Figure 13. Distribution of Hyper-active and Hypo-active significant drivers in BRCA data.** The drivers were separated by activity state (Hyper/Hypo), driver class (Overt vs Hidden), and type [TF (blue), SIG (orange), and NC (green)]. Each bar represents the number of drivers in the corresponding category, with stacked colors indicating driver type.

**Supplementary Figure 14.** Heatmap depicting the weight correlations between pathways (connected to at least three driver genes) and top SHAP values from each model that connected to at least one pathway for the BRCA case study.

**Supplementary Figure 15.** **Submodule network representation of lncRNAs key driver hubs for BRCA case study.** This figure shows (A) the GATA3-AS1/DSCAM-AS1 Co-Hub Module and (B) the AFAP1-AS1 Hub Module. Nodes represent functional categories [non-coding RNAs (yellow), signalling molecules (blue), transcription factors (red), and pathways (green)] with edges indicating regulatory interactions.
