# Supplementary material for: lncAPNet enables the deciphering of lncRNA–mRNA connections in patient transcriptomic data
Source: Bioinform Adv. 2026 Jul 21;6(1):vbag204. doi: 10.1093/bioadv/vbag204 (PMC13485353; doi:10.1093/bioadv/vbag204)
Supplement: Supplementary file 1 [file vbag204_supplementary_data.zip › Supplementary_Tables.docx]

**Supplementary Table 1.** Summary of Gene Associations in CLL case study with Observed Average Distances Between lncRNAs Identified in Studies and Experimentally Validated lncRNAs from the lncHUB UMAP.

| **Case Study** | **Gene** | **Obs. Avr Distance** | **P-value (Closeness)** |
| --- | --- | --- | --- |
| ***CLL*** |  | | |
|  | FIRRE | 0.332 | **0.0150** |
|  | LINC00158 | 11.221 | 0.9560 |
|  | KCNJ2-AS1 | 3.926 | 0.2060 |
|  | FLNB-AS1 | 2.156 | 0.1060 |

**Supplementary Table 2.** Summary of Gene Associations in PRAD case study with Observed Average Distances Between lncRNAs Identified in Studies and Experimentally Validated lncRNAs from the lncHUB UMAP.

| **Case Study** | **Gene** | **Obs. Avr Distance** | **P-value (Closeness)** |
| --- | --- | --- | --- |
| ***Prostate***  ***Adenocarcinoma*** |  | | |
|  |  |  |  |
|  | PRC1-AS1 | 0.200 | **0.0210** |
|  | FRY-AS1 | 0.751 | 0.2150 |
|  | LINC01082 | 0.284 | **0.0400** |
|  | HCG11 | 1.101 | 0.3830 |
